# Genome wide association study unveils the genetic basis of *Orobanche crenata* resistance in pea

**DOI:** 10.1007/s00122-025-05051-2

**Published:** 2025-10-11

**Authors:** Osman Zakaria Wohor, Nicolas Rispail, Diego Rubiales

**Affiliations:** 1 Institute for Sustainable Agriculture, CSIC, Avda. Menéndez Pidal S/N, 14004 Cordoba, Spain; 2 Savanna Agriculture Research Institute, CSIR, Post Office Box TL52, Nyankpala, Tamale, Ghana

## Abstract

**Key message:**

GWAS using DArTseq markers identified novel resistance sources against parasitic broomrape in pea, elucidating candidate genes for marker-selected breeding as leverage for cultivar development and efficient disease control to enhance food security.

**Abstract:**

Crenate broomrape (*Orobanche crenata*) is an important obligate root parasitic weed that causes severe yield losses in pea (*Pisum sativum*) production. *O. crenata* is difficult to eradicate in pea fields due to its high resilience and prolific seed boom capable of hibernating in soils for decades. Existing control strategies are not cost effective in low input legumes like pea. The most efficient ecofriendly mode of control is using resistant cultivars. Quantitative trait loci (QTL) studies based on bi-parental mapping has guided *O. crenata* resistance discovery, albeit their deployment in pea breeding is hindered by low marker resolution and large genetic distance. This study presents the first genome-wide association study (GWAS) on *O. crenata* resistance in pea, utilizing 324 diverse accessions and 26,045 diversity array technology sequence (DArTseq) markers. Phenotyping was performed over four seasons under field conditions using alpha lattice design. Results showed a strong phenotypic variation with an environmental influence on *O. crenata* infection. Novel resistance sources were identified mainly within the wild *Pisum fulvum* and *P. sativum* subsp. *elatius.* GWAS with two models yielded a total of 73 marker-trait associations with Chromosome 5 as major hotspot. Interestingly, some linked markers were detected in close proximity to four previous *O. crenata* resistance QTL. DArTseq markers identified 24 putative candidate genes participating in different cellular processes, including vesicle trafficking and transports, deoxyribonucleic acid transcription regulation, and defense including some leucine rich repeat receptor-like kinases. These results provide a valuable genetic resource for *O. crenata* resistance and a step toward its effective sustainable management—to enhance genetic diversity and cultivar improvement for food security.

**Supplementary Information:**

The online version contains supplementary material available at 10.1007/s00122-025-05051-2.

## Introduction

Pea (*Pisum sativum* L.) is a self-pollinated diploid species (2*n* = 14) with a haploid genome size of 4.5 Gbp (Smýkal et al. 2012). Globally, pea is the second most widely cultivated legume, following soybean. Dry pea occupies approximately 7.2 million hectares, producing an average yield of 2 tons per hectare. Green peas, on the other hand, are cultivated on about 2.6 million hectares with a significantly higher average yield of 8 tons per hectare (FAOSTAT, 2024). Pea is an economical and versatile protein source for animal feed and human diets, it maintains soil fertility, and it is increasingly used in the food industry (Rubiales et al. 2020). However, in Mediterranean farming systems, pea cultivation is heavily constrained by crenate broomrape (*Orobanche crenata* Forsk.) (Rubiales 2023). *O. crenata* is an obligate soilborne root parasitic weed known to cause severe damage or complete yield loss in several legume crops (Parker 2013). *O. crenata* germination and parasitic association is instigated by host root exudates and nutrient uptake is exclusively within host vascular root systems (Pérez-de-Luque et al. 2009). The interspecific parasitic association between pea and *O. crenata* hinders the effectiveness of existing cultural, biological, and integrated control methods, including the use of selective herbicides (Rubiales et al. 2009a; Rubiales and Fernández-Aparicio 2012). Consequently, genetic resistance is considered the most effective and sustainable broomrape management strategy (Rispail et al. 2007; Rubiales 2018). However, many genetic factors governing the pea-*O. crenata* association remain unknown. In addition, the limited resistance in existing pea cultivars detected so far with only some level of partial resistance attained and its low heritability had hindered the development of commercially viable *O. crenata* resistant cultivars (Rubiales et al. 2006, 2009b; Pavan et al. 2016). Response to *O. crenata* involves the interaction of multiple resistance mechanisms acting at different stages of the infection process, which include escape due to earliness or reduced root biomass, low germination induction, or post-attachment inhibition among other traits. These mechanism could be due to increased protein cross-linkage, or enhanced activity of peroxidase and/or *β*-1, 3-endoglucanase (Rubiales 2003; Pérez-de-Luque et al. 2005, 2006; Lozano-Baena et al. 2007; Fernández-Aparicio et al. 2012; Pavan et al. 2016). However, these traits have been insufficiently studied at the molecular level.

Resilient cultivars can be achieved by pyramiding valuable agronomic traits with disease resistance through effective deployment of pea genetic resources (Rubiales et al. 2023). Pea landraces and wild relatives are excellent potential genetic reservoirs for identifying *O. crenata* resistance genes absent in pea cultivars (Rubiales et al. 2005; Pérez-de-Luque et al. 2009; Rubiales 2023). Conventional breeding by crossing and selection has succeeded in the development of advanced breeding lines with moderate to high levels of resistance, but the process is slow and tedious (Rubiales et al. 2009b, 2020; Fondevilla et al. 2017). The advent of high-throughput genotyping and reduction of sequencing cost, coupled with the availability of annotated pea reference genomes (Kreplak et al. 2019; Yang et al. 2022; Liu et al. 2024), offer a unique opportunity to unravel pea–*O. crenata* interaction using omics and mapping studies (Pandey et al. 2021; Parihar et al. 2022; Wohor et al. 2022). Nevertheless, prior to these recent advancements, QTL mapping of bi-parental populations successfully identified molecular markers linked to *O. crenata* resistance in pea (Valderrama et al. 2004; Fondevilla et al. 2010; Delvento et al. 2023). Similarly, other studies reported expressed genes (Die et al. 2009) or proteins (Castillejo et al. 2004, 2012) related to defense response to *O. crenata* in pea. In the model legume *Medicago truncatula* proteomic studies identified a number of functional genes (Dita et al. 2009) and proteins (Castillejo et al. 2009) potentially linked with resistance to *O. crenata*.

Quantitative trait loci (QTL) mapping can determine phenotypic differences influenced by a few genes with strong effects or many genes with minor effects (Myles and Wayne 2008). The low level and highly quantitative broomrape resistance observed in pea suggested the implication of several minor genes each explaining a small portion of the phenotypic variation. QTL mapping in pea inbred lines identified two QTLs (*Ocp1* and *Ocp2*) significantly linked to *O. crenata* resistance, which together explained 20% of phenotypic variance (Valderrama et al. 2004). Population of recombinant inbred lines (RIL) were also used to elucidate four QTLs (*nºbr03_1, nºbr03_2, nºbr03_3*, and *nºbr04_1*) associated with *O. crenata* resistance, explaining between 10 and 17% of the phenotypic variance for resistance assessed under field studies and between 8 and 37% under controlled evaluation in mini-rhizotrons (Fondevilla et al. 2010). Subsequently, three additional QTLs (*PsOcr-1, PsOcr-2, PsOcr-3*) were mapped in a different bi-parental population assessed under field conditions (Delvento et al. 2023).

Therefore, QTL mapping has been informative, but its precision is limited to the restricted genetic diversity within bi-parental segregants and limited genetic markers, which hinder gene discovery (Myles and Wayne 2008). Genome-Wide Association Studies (GWAS) can complement traditional QTL mapping to accelerate gene discovery and overcome the constraints associated with bi-parental mapping (Myles and Wayne 2008; Bush and Moore 2012). GWAS based on linkage disequilibrium (LD) is used to extensively profile natural variations in large diverse germplasm to identify maker-trait associations (Crosta et al. 2023). Association mapping has significantly reduced genome complexity and advanced the identification of single nucleotide polymorphisms (SNPs) corresponding to active genes (Thudi et al. 2011; Crosta et al. 2023). Diversity array technology sequencing (DArTSeq) can deliver large cost effective molecular markers with broad coverage in whole genome fingerprinting (Jaccoud et al. 2001; Barilli et al. 2018; Rispail et al. 2023), suitable for GWAS. In pea, GWAS guided the identification of variant-trait associations for breeding valuable biotic stress resistance and quality traits (Desgroux et al. 2018; Gali et al. 2019; Osuna-Caballero et al. 2024a, 2024b; Uhdre et al. 2024). Recently, GWAS revealed candidate genes linked to resistance to the parasitic weed *Striga gesneroides* in cowpea (Koura et al. 2024), and also elucidated resistance to *S. hermonthica* in sorghum (Kavuluko et al. 2021) as well as in maize (Dossa et al. 2024). These insights are valuable for enhancing genetic resistance against other parasitic weeds like *O. crenata*.

Advanced GWAS techniques can enhance trait associations to narrow the genetic distance between flanking markers and alleles for marker assisted selection (MAS). However, GWAS-based investigations of pea resistance to *O. crenata* remain a significant knowledge gap. Accordingly, we evaluated a diversity panel of 324 *Pisum* accessions over four cropping seasons under field conditions and performed a GWAS analysis using DArTseq markers. This study aims to provide DArT-markers associated with *O. crenata* resistance and unveil the underlying genes and pathways conferring resistance to this parasitic weed. Integrating *O. crenata* resistance into breeding programs can significantly expand genetic diversity, leading to the development of improved cultivars to deliver sustained genetic gains and enhance food security.

## Materials and methods

### Plant material

This study used a diverse core collection composed of 324 accessions carefully selected to represent the *Pisum* genera in terms of taxonomy, geographic distribution, and phenotypic variation. Taxonomic classification of the accessions was based on previous phylogenetic and genetic diversity assessment of the collection (Rispail et al. 2023) and followed the GRIN taxonomy classification (GRIN Global 2025). Accordingly, the collection was composed of 13 accessions of *P. fulvum*, 6 of *P. abyssinicum*, 20 of *P. sativum* subsp. *elatius* var. *elatius,* 26 of *P. sativum* subsp. *elatius* var. *pumilio*, 78 of *P. sativum* subsp. *jomardii,* 94 of *P. sativum* subsp. *sativum* var. *arvense,* 66 of *P. sativum* subsp. *sativum* var. *sativum* and 21 of the “Indian ecotype” of *P. sativum* subsp. *sativum* (Supplementary table S1). In addition, the collection included accessions previously described as partially resistant to *O. crenata* (Rubiales et al. 2005, 2020). The pea diversity panel was assembled and maintained at the Institute for Sustainable Agriculture (IAS)–CSIC in Cordoba, Spain. The panel was initially curated from a large *Pisum* spp. collection of > 3000 accessions provided by USDA (Department of Agriculture, Pullman, WA, USA), JIC (John Innes Center, Norwich, UK), CRF (Centro Nacional de Recursos Fitogenéticos, Madrid, Spain), CGN (CPRO-DLO, Wageningen, The Netherland), IPK (Leibniz Institute of Plant Genetics and Crop Plant Research, Gatersleben, Germany) and ICARDA (International Center for Agricultural Research in the Dry Areas, Aleppo, Syria).

### Field experimental setup and broomrape assessment

The IAS pea diversity panel was screened for broomrape resistance at Cordoba, Spain in a heavily infested field over four cropping seasons (2017/2018, 2018/2019, 2019/2020 and 2020/2021) under rainfed conditions. These four seasons are hereby represented as Cor18, Cor19, Cor20, and Cor21. Soil dynamics and rainfall parameters were also considered. Average minimum and maximum temperatures during the growing seasons ranged from 6 to 9 °C and 20 to 22 °C, respectively (Table 1). Pea accessions were sown in 1-m rows of 10 seeds, arranged in an alpha lattice design with three replications. A row of the susceptible check cultivar Cartouche, was included in every two experimental rows. Distance between adjacent rows was 0.7 m. To ensure optimal germination and homogeneity in seedling emergence, all pea seeds were scarified before sowing. Broomrape seeds were collected locally from previous pea fields, mixed with sand at a density of 1 g per m^2^, and evenly spread underneath each row to ensure homogeneous infection. Experimental plots were planted in November/December each year, to take advantage of the early spring rains to ensure optimal broomrape emergence. Recommended pea agronomic management practices were strictly followed, according to local practices, excluding pre-emergence herbicides. Weeding was performed by hand to avoid interfering with broomrape development. The number of emerged *O. crenata* per plant (*N*_BR_p_) was determined at full bloom and the crop harvested during the summer (May–June) (Rubiales et al. 2006).

**Table 1:** Description of edaphic properties in the multi-environment trials during the pea growing cycle, from sowing (November–December) to harvest (June)

ENV	Season	Site (decimal degrees coordinates)	Soil Type	Soil pH	Organic Matter (g/100 g)	Available Phosphorus (mg/kg)	Average T_max_ (°C)	Average *T*_min_ (°C)	Rain (mm)
Cor18	2017–2018	37.860721	Cambisol	–	–	–	19.6	7.3	448
		− 4.798860							
Cor19	2018–2019	37.860670	Cambisol	7.5	1.2	15.1	21.8	6.3	127
		− 4.799171							
Cor20	2019–2020	37.860492	Cambisol	–	–	–	21.8	9.0	348
		− 4.799375							
Cor21	2020–2021	37.860585	Cambisol	7.7	1.1	12.1	21.3	8.2	341
		− 4.799192							

### Data management and statistical analysis

To account for potential heterogeneity in broomrape infestation across experimental fields, N_BR_p_, data were standardized by adjusting values based on the deviation of Neighboring susceptible check (N_check_) plots from the overall susceptible mean check (Mean_check_) with the formula:

$${\mathrm{N}}_{{{\mathrm{BR}}\_{\mathrm{p}}}} = {\mathrm{N}}_{[{\mathrm{i}}]} \times \frac{{{\mathrm{Mean}}_{{{\mathrm{check}}}} }}{{\frac{{\left( {{\mathrm{N}}_{{{\mathrm{check[i} - 1]} }} + {\mathrm{N}}_{{{\mathrm{check[i} + 1]} }} } \right)}}{2}}}$$

where N_[i]_ is the N_BR_p_ of the accession in the *i*th row, Mean_Check_ is the general N_BR_p_ mean of the check accession and N_Check[i−1]_ and N_Check[i+1]_ are the N_BR_p_ of the check plots located in the accession’s adjacent rows.

The outliers that represent N_BR_p_ values below the 5% percentile or higher than the 95% percentile were removed from the dataset and the missing data were imputed with the classification and regression trees imputation (cart) method (Breiman et al. 2017). Outlier trimming and data imputation were performed in R (R Core Team 2024), for each accession and environment using the ‘mice’ and ‘miceadds’ R packages (Van Buuren and Groothuis-Oudshoorn 2011; Robitzsch et al. 2017). Data quality was further examined on the resulting dataset, through graphical inspection of residuals to assess outliers, normality, and homogeneity of variance. Deviation from normality and homogeneity of variance were also assessed with the Shapiro–Wilks and Levene tests, respectively, in R using the “rstatix” package (Kassambara 2023). Preliminary analysis indicated homogeneous variances (*p* = 1) but detected deviation of normality (*p* = 2.76e^−49^). Accordingly, the N_BR_p_ data was subjected to square root transformation to normalize residuals. Then, linear mixed models were applied to analyze the data. Data collected for each environment were analyzed individually with a one-way mixed effect model following the formula:

$$y_{ijk} = \mu + \alpha_{i} + \gamma_{j} + \left( {\gamma \tau } \right)_{jk} + \varepsilon_{ijk}$$

where ${y}_{ijk}$ is the *N*_BR_p_ value of the *i*th accession (*i* = 1, 2, …, 324) in the *k*th incomplete block (*k* = 1, 2, …, 18) of the *j*th replicate (*j* = 1, 2, 3); ${\alpha }_{i}$ is the random effect of the *i*th accession;${\gamma }_{j}$ is the fixed effect of the *j*th complete replicate; ${(\gamma \tau )}_{jk}$ is the random effect of the *k*th incomplete block nested within the *j* replicate; and ${\varepsilon }_{ijk}$ is the error associated to ${y}_{ijk}$. Broad-sense heritability (*H*^2^) was estimated as the ratio of genotypic variance to phenotypic variance (Toker 2004). Subsequently, a joint analysis (joint-BLUP) was performed by applying a multi-environment trial (MET) two-way linear mixed model with interaction effect on each environmental data using the formula:

$$y_{ilk} = \mu + \alpha_{i} + \tau_{l} + \left( {\alpha \tau } \right)_{lk} + \gamma_{lk} + \varepsilon_{ilk}$$

In this case, ${\tau }_{l}$ is the fixed effect of the *l*th environment ${(\alpha \tau )}_{lk}$ is the interaction random effect of the *i*th genotype with the *l*th environment;${\gamma }_{lk}$ is the fixed effect of the *k*^th^ block within the *l*th environment. In this model, genotype, genotype-environment interaction, and incomplete blocks nested within complete replicates were considered as random effects while environments and the complete replicates nested within environments were assumed as fixed effect. Heritability in the MET model was calculated as the genotypic variance divided by the sum of the genotypic variance, genotype-by-environment interaction (GEI) variance, and residual variance. In both cases, restricted maximum likelihood (REML) procedure was used to estimate variance components of the models and to compute the best linear unbiased prediction (BLUP) (DeLacy et al. 1996). BLUPs were used as phenotypic data for all subsequent analysis. Mixed linear models and BLUP estimation was performed in R using the “metan” package (Olivoto and Lúcio 2020).

To delve deeper into GEI and identify the most resistant accessions across environments, the dataset was further analyzed through a genotype-genotype-by-environment (GGE) biplot approach (Yan and Holland 2010). This approach estimates the major principal components (PCs) based on symmetric scaling and singular value decomposition (SVD) of MET data. This model uses the first two PCs to build a biplot for the simultaneous visualization of genotypic performance and environmental interaction, that facilitate the identification of superior genotypes and classification of environments (Yan et al. 2007). To ease visualization of the most resistant pea accessions, data used to draw the biplot was genotype-centered without scaling. The GGE model was estimated using the “metan” package in R.

### Genotyping

The method for DNA extraction, library preparation and sequenced data analysis procedure were previously described in Rispail et al. (2023). Briefly, the 324 accessions composing the IAS pea diversity panel were genotyped by DArTseq, using DNA samples extracted from leaves of a week-old seedlings (20 per accession) and analyzed using the high-density Pea DArTSeq 1.0 array. Upon assembly, low quality and non-polymorphic markers were removed from the dataset providing a total of 26,045 Silico-DArT markers and 11,511 SNPs, which were aligned to the Caméor and ZW6 pea reference genomes (Kreplak et al. 2019; Yang et al. 2022). Since Silico-DArT markers provided higher genome coverage and smaller linkage disequilibrium decay distance than SNPs (Rispail et al. 2023), this dataset was used for GWAS.

### Population structure

Prior to estimating the population structure of the IAS pea diversity panel, markers in significant LD were filtered from the SilicoDArT dataset. LD filtering was performed with the pruning method implemented in PLINK 1.9 (Chang et al. 2015) with a window size of 200 markers and an r^2^ threshold of 0.1. Then the LD filtered SilicoDArT dataset (3,465 markers) was analyzed with ADMIXTURE 1.3.0 (Alexander et al. 2009) that implements a maximum-likelihood model-based approach. ADMIXTURE analysis consisted of 10 independent simulations using different starting point for each number of k ranging from 1 to 25. Each simulation was stopped once the convergence criterion (Δ) was below 0.0001. Cross-validation test with 10 iterations was performed for each simulation. The optimal number of k was then determined according to the cross-validation error for each k and through estimation of the Δ*k* (Evanno et al. 2005). The corresponding Admixture Q matrixes was then obtained and visualized with the online software CLUMPAK (Kopelman et al. 2015).

### Genome-wide association studies

To identify molecular markers associated with resistance to *O. crenata*, the Silico-DArT dataset was combined with field-based phenotypic dataset and analyzed by GWAS. GWAS was done under “GAPIT 3.0” R package (Wang and Zhang 2021) using two different models: Fixed and random model Circulating Probability Unification (FarmCPU) (Liu et al. 2016), and Bayesian information and Linkage-disequilibrium Iteratively Nested Keyway (BLINK) (Huang et al. 2019). GWAS models incorporated the Astle kinship matrix that estimates the relatedness between individuals to account for potential genetic correlations (Astle and Balding 2009) and the first three PCs as covariates to ensure optimal correction of population structure. Efficient control of population structure of the pea collection was checked by estimating the genomic inflation factor (*λ*) (Devlin et al. 2001) and by visual examination of the resulting quantile–quantile (QQ) plots. All models showing significant genomic deflation or inflation with *λ* values below 0.8 or higher than 1.2, respectively, were discarded from the analysis (Reed et al. 2015). Genomic inflation factor was estimated with the R package “QQperm” (Petrovski and Wang 2016). Two significance thresholds, the stringent Bonferroni correction limit and the less restrictive false discovery rate were applied to ensure control of false positives during selection of significant marker-trait associations (MTAs). FDR threshold was estimated for each model with the R package “Qvalue” (Storey and Bass 2024) and set below one false positive. Manhattan plots displaying the -log10 (p-value) for each marker were used for graphical observation of significant MTA. All unaligned markers were arbitrarily included on an artificial ninth chromosome (chr9) on the Manhattan plot to ease result visualization. Manhattan and QQ plots were generated with the “CMplot” R package (Yin 2023).

### Candidate genes and pathways selections

To detect potential candidate genes linked to significant MTA, the genomic regions surrounding these markers were examined within a 30 kb window using the genome browsers of the Caméor and ZW6 pea reference genomes (Kreplak et al. 2019; Yang et al. 2022), implemented in the Pulse Crop Databases (https://www.pulsedb.org/jbrowses). Candidate gene annotation was then performed by BLAST (Altschul et al. 1990) using *blastn* and *blastx* algorithm implemented in the NCBI BLAST webserver (Johnson et al. 2008; Accessed on 24/06/2024) on the NCBI *nr* database. InterPro database (Blum et al. 2021) was also used to search for the presence of potential functional domains in the deduced protein sequences of the putative candidate genes with InterProScan 5 (Jones et al. 2014). To circumvent potential discrepancies between Caméor and ZW6 reference genomes, BLAST and INTERPROSCAN annotations was performed on the annotated sequence of the candidate genes of both reference genomes yielding similar results in all cases.

## Results

### Phenotypic evaluation of the IAS pea diversity panel against *O. crenata*

**Fig. 1:**
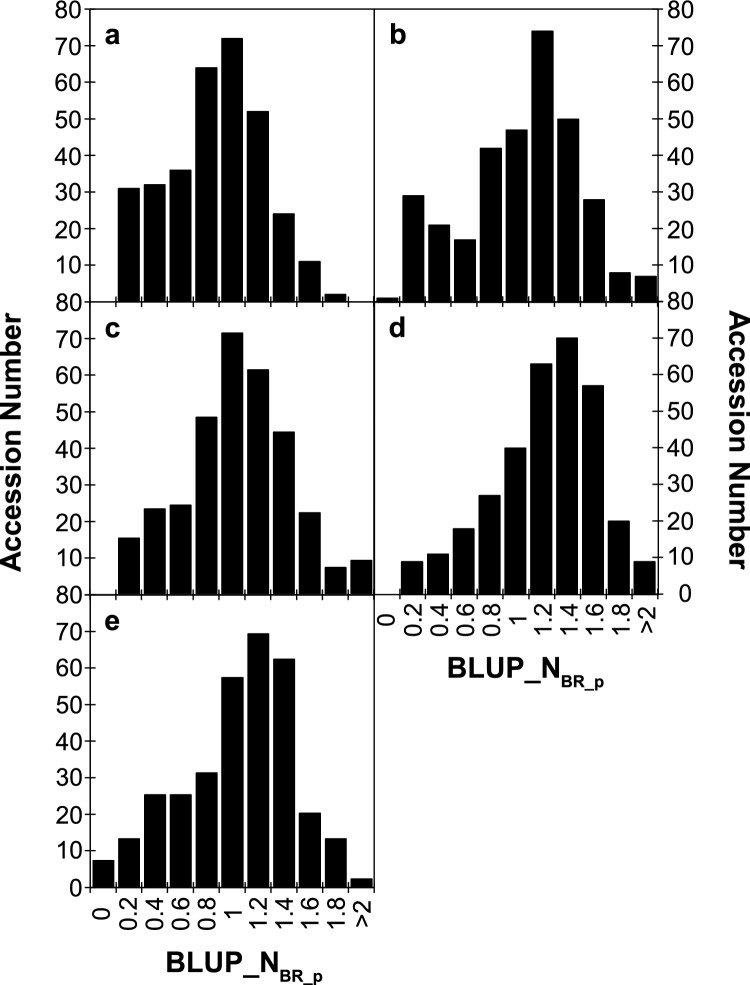
Distribution of accessions showing quantitative response to broomrape (BLUP_N_BR_p_) under field conditions. The histograms represent the distribution of the response of the pea diversity panel for the four environments, Cor18 (**a**), Cor19 (**b**), Cor20 (**c**), Cor21 (**d**) and the Joint environment (**e**)

All pea accessions showed affinity to infection by broomrape under field conditions although large phenotypic variation was observed from relatively low to very high levels of broomrape infection (Table 2 and Fig. 1). Distribution of BLUP_N_BR_p_ across the diversity panel showed a positive and moderate skewness. Differences in the overall infection level was observed between environments. Cor18 presented the lowest level of infection varying from 0 to 9.64 BLUP_N_BR_p_ with a mean value of 1.34 BLUP_N_BR_p._ Cor21 was the most heavily infected season, ranging from 0 to 11.29 with a mean of 2.13 BLUP_N_BR_p_. Heritability of N_BR_p_ was moderate, varying from 0.31 for Cor18 to 0.5 for Cor21 indicating that it is strongly influenced by environmental factors. This is also supported by the genomic variation coefficient (CV_g_) that varied from 53.5 to 67.6 from Cor21 and Cor20, respectively. Despite the strong influence of environment, the coefficient of determination (*r*^2^) of GEI explained only 4.5% of the phenotypic variance indicating weak genotype-environment interaction (*r*_ge_ = 0.091) (Table 2). Accuracy of the linear mixed model used to estimate individual and joint BLUP values varied from 0.76 for Cor18 to 0.96 for the joint environment (Joint_ENV) confirming the goodness of fit and adequacy of such approach for studying the phenotypic variation of the pea collection against *O. crenata* under varying field conditions.

**Table 2:** Descriptive statistics of BLUP_N_BR_p_ variation for each environment and the joint analysis

	Cor18	Cor19	Cor20	Cor21	Joint_ENV
Mean	1.34	1.73	1.68	2.13	1.72
SE	0.047	0.050	0.052	0.052	0.026
Min	0	0	0	0	0
Max	9.64	8.9	9.95	11.29	11.29
Skewness	1.94	1.41	1.91	1.27	1.58
H^2^	0.31	0.45	0.49	0.50	0.50
Accuracy	0.76	0.85	0.86	0.86	0.96
CVg	61.13	61.31	67.60	53.53	39.10
CV ratio	0.67	0.92	0.99	0.99	1.06
GEI *r*^*2*^					0.045
*r*_ge_					0.091

**Fig. 2:**
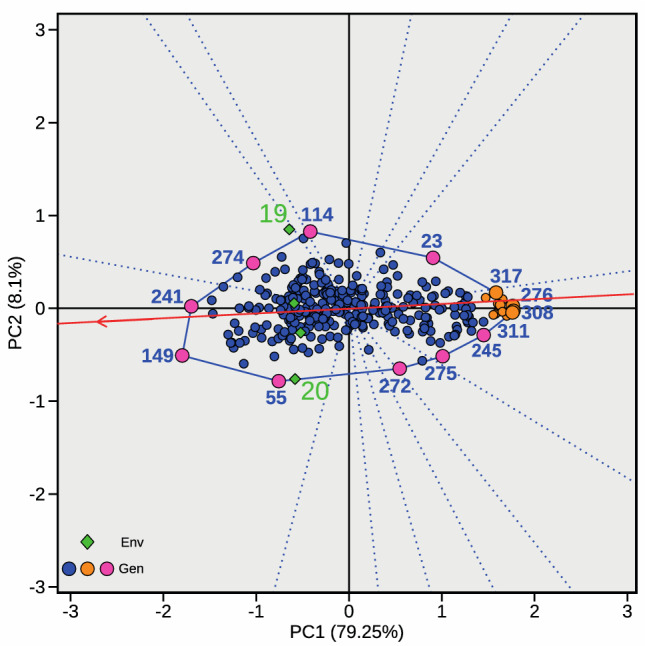
Genotype by Genotype by Environment interaction biplot. The graph represents the which-won-where biplots centered on the genotypes to facilitate observation of the most responsive pea accessions to the environments for their resistance to broomrape. The average environment vector (TEA) is represented on the biplot as a red arrow while individual environment vectors are represented by green diamonds. Pea accessions are represented on the biplot as blue dots except the most resistant accessions that are represented by orange dots. The most responsive accession to environment that define the GGE biplot polygon vertex are represented by pinkish dots

GGE biplot approach was applied to the estimated BLUP dataset to thoroughly investigate the GEI and categorize the pea accessions’ response to *O. crenata*. The first two PCs represented 87.35% of the total BLUP_N_BR_p_ variation, where PC1 accounted for 79.25% of the variation and PC2 for 8.10%. The which-won-where biplot placed the environment vectors (represented by green diamonds) on the left of PC1 (Fig. 2). The environment average (TEA) which is the key reference for interpreting genotype performance relative to the overall environmental mean was also oriented toward the left quadrants of the GGE biplots and only slightly departed from the biplot origin supporting the relatively low GEI (Fig. 2). Accordingly, most genotypes (represented by blue dots) are located near the origin revealing their low responsiveness to environment in terms of broomrape emergence. Accessions observed farther away from the origin are more responsive to environmental changes. The most responsive accessions to the environments were the accessions #149, #241, #274, #114, #23, #317, #276, #308, #311, #245, #275, #272 and #55 that defined the vertex of the biplot polygon (Fig. 2). Among them, accessions #149, #241, #55, #274 and #114, located on the left quadrant in the direction of the environment vectors were the most susceptible accessions to broomrape in at least one environment while accessions #23, #272, #275, #317, #276, #308, #311 and #245 located on the right quadrant and opposite the environment vectors were the more resistant accessions in at least one environment (Fig. 2). Interestingly, accessions #317, #276, #308 and #311 located on the polygon vertex opposite the vector environment and closest to the TEA vector were the most resistant accessions across environments. The accessions with lowest BLUP_N_BR_p_ value and stability across environments (represented as orange dots in Fig. 2) are shown in Table 3 while the full dataset is presented in Supplementary Material (Supplementary Table S1).

**Table 3:** Passport information and mean number of broomrapes per plant (N_BR_p_) values for the most resistant and susceptible accessions by species (spp.) and subspecies (subsp.) to broomrape under field condition

Accession	Passport Nº	Spp./Subsp.	Cor18	Cor19	Cor20	Cor21	Joint_ENV
Most resistant accessions							
308	IFPI 3253	*P. fulvum*	0.00	0.00	0.03	0.00	0.01
276	PIS 1318/91	*P. sativum* subsp*. elatius* var. *elatius*	0.00	0.00	0.00	0.04	0.01
311	IFPI 3261	*P. fulvum*	0.00	0.00	0.05	0.00	0.01
109	JI 224	*P. fulvum*	0.00	0.03	0.00	0.09	0.03
318	IFPI 3358	*P. sativum* subsp. *jomardii*	0.03	0.03	0.03	0.05	0.03
95	PI 560065	*P. fulvum*	0.00	0.00	0.05	0.10	0.04
305	IFPI 3232	*P. fulvum*	0.10	0.00	0.06	0.00	0.04
316	IFPI 3334	*P. sativum* subsp*. elatius* var. *elatius*	0.08	0.03	0.00	0.08	0.05
54	PI 343993	*P. sativum* subsp. *jomardii*	0.00	0.00	0.00	0.19	0.05
312	IFPI 3262	*P. fulvum*	0.00	0.00	0.07	0.19	0.06
317	IFPI 3338	*P. sativum* subsp*. elatius* var. *elatius*	0.09	0.16	0.00	0.03	0.07
113	JI 185	*P. sativum* subsp. *jomardii*	0.01	0.09	0.04	0.18	0.08
314	IFPI 3282	*P. sativum* subsp*. elatius* var. *elatius*	0.00	0.11	0.22	0.00	0.08
96	PI 560067	*P. fulvum*	0.00	0.07	0.30	0.00	0.09
Most susceptible accessions							
321	Cartouche	*P. sativum* subsp. *sativum* var. *sativum*	0.11	2.29	2.14	2.05	1.65
155	BGE026429	*P. sativum* subsp. *sativum* var. *arvense*	3.89	4.52	3.66	6.56	4.66
241	BGE025727	*P. sativum* subsp. *jomardii*	4.00	5.66	5.48	5.69	5.21
149	BGE020326	*P. sativum* subsp. *sativum* var. *arvense*	4.56	4.69	8.77	4.86	5.72

**Fig. 3:**
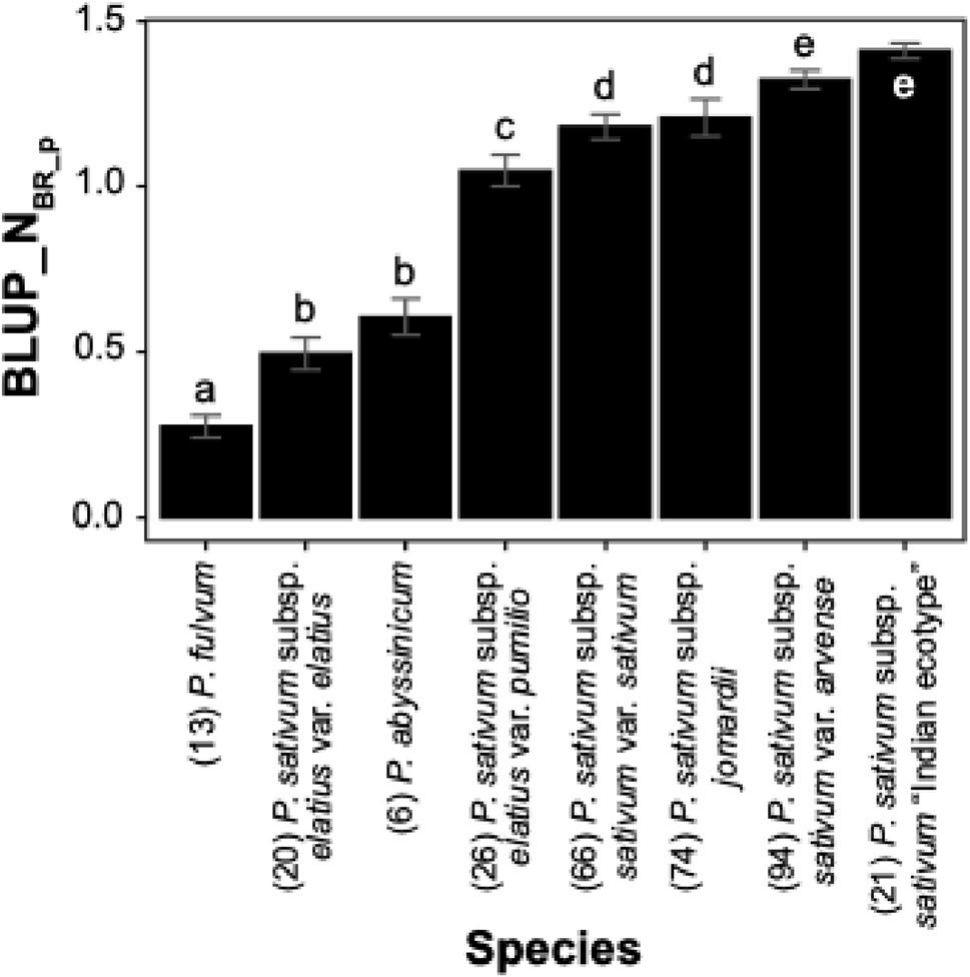
Response of the pea diversity panel to broomrape under field condition. The histogram represents the average (BLUP_N_BR_p_) values of number of broomrapes per plant for each *Pisum* species and subspecies in the joint environment. Letters on top of each bar indicate the significant grouping. Horizontal bars are standard error

Interestingly, wild accessions of *P. fulvum* and *P. sativum* subsp. *elatius* var. *elatius* exhibited the highest levels of resistance to *O. crenata* (Table 3). To further delve into the association between accessions and broomrape susceptibility, the average of the estimated BLUP_N_BR_p_ values were examined after grouping pea accessions based on their *Pisum* species and subspecies (Fig. 3). This revealed that, *P. fulvum, P. abyssinicum* and *P. sativum* subsp. *elatius* var. *elatius* accessions supported a smaller number of broomrapes than the remaining *P. sativum* subspecies. This information can be valuable for selecting broomrape resistant parents for pea improvement.

### Population structure of the IAS pea diversity panel

**Fig. 4:**
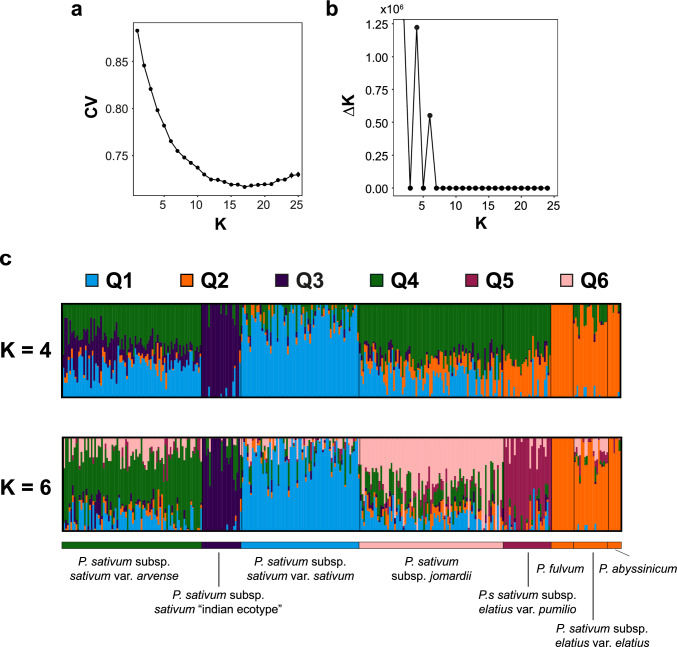
Population structure of the IAS pea diversity panel. **a** Evolution of the cross-validation error per *K* clusters. The graphic represents the mean cross-validation coefficient estimated by ADMIXTURE. Horizontal bars are standard error for *n* = 10. **b** Evolution of the Evanno’s parameter Δ*K* estimated per *K* clusters. **c** ADMIXTURE output for K = 4 or 6. Bars of the histograms represent the percentage of membership to each ADMIXTURE subpopulation of each pea accession

Prior to GWAS implementation, the population structure of the diversity panel was established with ADMIXTURE. The lowest CV error was obtained for *K* = 17 which did not allow assigning genotype to discrete subpopulations but rather as highly admixed accessions (Fig. 4a). Visual assessment of the evolution of CV error per cluster shows a shift of the curve at *K* = 6, favouring the presence of 6 subpopulations. Since observation of the cross-validation procedure did not allow easy detection of the optimal number of *K*, the Evanno parameter (Δk) was also estimated showing two peaks at *K* = 4 and *K* = 6 (Fig. 4b). For *K* = 4, four distinct groups emerged: Q1, comprising of the cultivated *P. sativum* subsp. *sativum* var*. sativum*; Q2, the wild pea relatives including *P. fulvum*, *P. abyssinicum*, and *P. sativum* subsp. *elatius* var. *elatius*; Q3, containing the ‘Indian ecotype’ of *P. sativum* subsp. *sativum*; and Q4, composed of admixed accessions of *P. sativum* subsp. *sativum* var. *arvense*, *P. sativum* subsp. *jomardii*, and *P. sativum* subsp. *elatius* var. *pumilio* (Fig. 4c). A similar genotypic assignment was observed for *K* = 6 that maintained Q1, Q2 and Q3 composition. However, the former admixed Q4 group was further divided into three highly admixed subpopulations corresponding to *P. sativum* subsp. *sativum* var. *arvense* (Q4), *P. sativum* subsp. *elatius* var. *pumilio* (Q5) and *P. sativum* subsp. *jomardii* (Q6).

### Detection of molecular marker associated with *O. crenata* resistance

Detection of molecular markers associated with broomrape resistance was performed by single and joint environment GWAS analysis. Although small variations of the genomic inflation (*λ*) factor were detected across models, *λ* was in all cases maintained within the 0.8–1.2 limits confirming that population structure was correctly controlled by the GWAS models (Fig. 4). Independently of the dataset, FarmCPU yielded the highest number of significant MTAs and explained the highest proportion of phenotypic variance (Table 3).

**Fig. 5:**
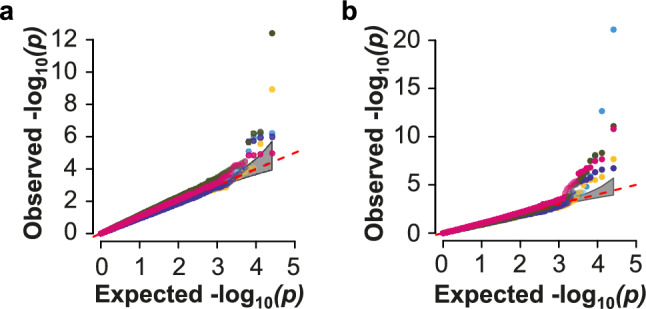
QQ plots of the **a** BLINK and **b** FarmCPU model outputs for broomrape resistance obtained for the individual and joint environments indicating the model adequacy within the threshold line (red dotted)

**Fig. 6:**
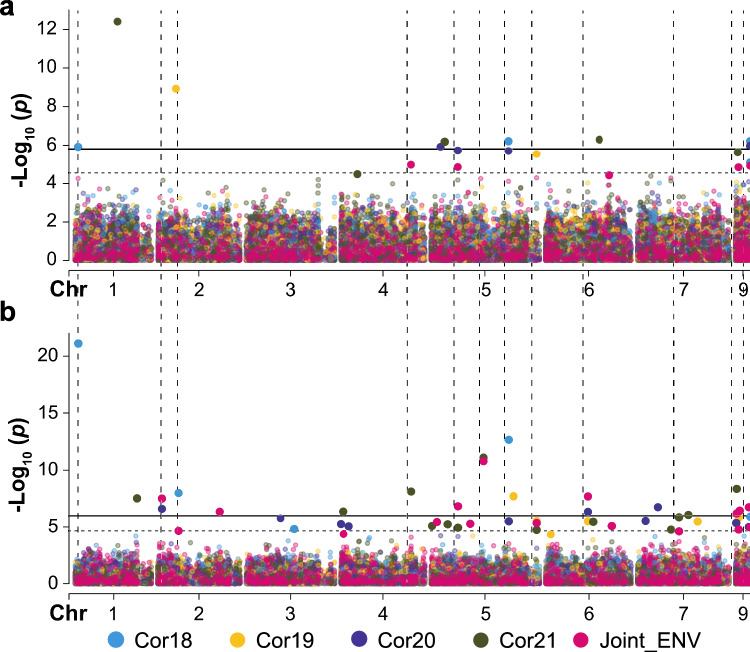
Superposed Manhattan plots displaying the *p* values of the DArT markers identified as significantly linked to broomrape resistance in the individual and joint environments by BLINK (**a**) and FarmCPU (**b**) models. The Manhattan plots show the marker-trait associations of the pea accessions in the multi-environment field trials. Horizontal black line and dashed gray lines represent the Bonferroni and FDR significance threshold respectively. All markers with *p-values* smaller than these thresholds were considered significant. Vertical gray dashed lines indicate MTA detected in several environments and/or GWAS models

Altogether the different models identified 73 significant MTAs corresponding to 50 SilicoDArT markers. Many of these markers were uncovered by a single model in one environment although 13 were detected in the two models and/or environments used. Analyzing the Joint_ENV that integrate the predicted BLUP_N_BR_p_ values of all tested environments yielded the highest number of MTAs. Most of these MTAs were also significant in a single environment (Table 4). Interestingly, 19 and 14 MTAs were detected in Cor21 and Cor20, respectively, that showed the highest broomrape infection pressure while only 9 and 8 MTAs were detected in Cor18 and Cor19, respectively. Significant MTAs were detected on all pea chromosomes highlighting the complexity of broomrape resistance. The highest number of significant MTAs were detected on chromosome 5 with a total of 22 significant associations corresponding to 12 SilicoDArT markers while only two MTAs were detected on chromosome 3 (Fig. 5 and Table 5). In addition, 17 significant MTAs, represented by 11 markers, could not be mapped to any existing pea reference genomes. In earlier studies, some putative QTLs (*B5/rl3, n*^*o*^*br3_2, PsOcr-3*) were found associated with broomrape resistance on chromosome 5 (Fondevilla et al. 2010; Delvento et al. 2023) These results suggest that chromosome 5 may be harboring a hotspot for stress response (Fig. 5). Interestingly, several of the SilicoDArT markers fall within the confidence interval of some previously identified QTL, including markers 5900,285 within QTL *nºbr04_1,* 3557002 within QTL *nºbr04_2*, 3543480 within QTL *td2,* the markers 5928200, 3642481 and 5934000 within QTL *B5/rl3* and the markers 3535900 and 5885796 within QTL *nºbr03_2* (Fig. 6).

**Table 4:** Summary of GWAS output obtained for broomrape resistance in the multi-environment field trial according to the BLINK and FarmCPU models

ENV	Model	Nº MTA	*λ*^a^	PVE_min_^b^	PVE_max_^b^
Cor18	BLINK	4	1	1.1	11.28
	FarmCPU	5	0.92	1.1	14.78
	Commojin	3			
Cor19	BLINK	2	0.98	1.2	8.28
	FarmCPU	6	0.84	2.13	46.9
	Common	1			
Cor20	BLINK	4	0.91	0.06	22.56
	FarmCPU	10	0.87	0.33	9.49
	Common	1			
Cor21	BLINK	6	1.2	1.1	19.28
	FarmCPU	13	0.97	0.05	26.34
	Common	0			
Joint_ENV	BLINK	6	1.07	1.16	46.55
	FarmCPU	16	1.08	0.54	24.93
	Common	2			
Total	Total	72			
	Unique	50			
	Common	13			

^a^*λ* is the genomic inflation factor, ^b^PVE_min_ and PVE_max_ is the minimum and maximum value of phenotypic variance explained by individual significant marker-trait associations (MTAs).

**Table 5:** List of significant markers identified by GWAS for the single and joint environment with the BLINK and FarmCPU models as associated with *O. crenata* resistance and related candidate genes with annotated pathways

Marker	Chromosome	Position	*P*. value	PVE	ENV_Model_	Gene ID_Caméor_	Gene ID_ZW6_	Distance (kb)	Gene annotation
5900285	1	17792641	1.22E−06	11.28	Cor18_BLINK_	Psat1g014200	Psat01G0028700	Inside	Myb-like domain protein
			7.82E−22		Cor18_FarmCPU_				
8053950	1	254913622	3.92E−13	7.66	Cor21_BLINK_	Psat0s3405g0080	Psat01G0293600	Inside	Tripeptidyl-peptidase 2 like protein
3560910	1	370024290	3.25E−08	2.45	Cor21_FarmCPU_	Psat1g222040	Psat01G0585700	8.38; 9.48	Serine Hydroxymethyl transferase like protein
3559439	2	24869503	2.66E−07	1.48	Cor20_FarmCPU_	Psat2g023160	Psat02G0073800	Inside	Calnexin like protein
			3.37E−08	0.54	Joint_ENV_FarmCPU_	Psat2g023200	Psat02G0073900	8.02; 8.13	Hsp20 /alpha crystallin domain containing protein
3551898	2	108338284	1.17E−09	1.21	Cor19_BLINK_	Psat2g062400	Psat02G0197400	Inside	60S ribosomal protein L10
4655918	2	123315717	1.12E−08	14.78	Cor18_FarmCPU_	Psat2g067800	Psat02G0218300	2.15	Plant 2-oxoglutarate-depdendent oxidoreductase related protein
			2.25E−05	14.79	Joint_ENV_FarmCPU_				
3566265	2	370846527	4.73E−07	17.11	Joint_ENV_FarmCPU_	Psat2g144320	Psat02G0417300	Inside	beta amylase 8
4656692	3	205367649	1.80E−06	9.49	Cor20_FarmCPU_	Psat3g104040	Psat03G0375800	Inside	BEACH domain-containing Protein
3560460	3	286800472	1.54E−05	12.22	Cor18_FarmCPU_	Psat3g148680	Not detected	Inside	Hyp prot containing peptidase S1 domain
3538710	4	574221	5.69E−06	2.05	Cor20_FarmCPU_	Psat4g001080	Not detected	0.17	Peptidoglycan biosynthesis/recognition domain containing Hyp prot
						Psat4g001040	Not detected	-0.41	NADPH-dependent pterin aldehyde reductase
3557002	4	13760470	4.60E−07	26.34	Cor21_FarmCPU_	Not detected	Psat04G0034300	29.5	Hyp Prot
3543480	4	46385029	8.94E−06	6.61	Cor20_FarmCPU_	Psat0s1953g0360	Psat04G0080700	Inside	Beta-glucosidase sfr2-like protein
8052497	4	98560812	3.34E−05	1.1	Cor21_BLINK_	Not detected	Psat04G0189200	− 0.794	Ser/thr Protein kinase like protein
						Psat4g059880	Psat04G0189100	− 4.11; − 5.72	Plant stress Response kinase and EF-hand domains containing Protein
3549925	4	419155842	1.09E−05	1.16	Joint_ENV_BLINK_	Psat4g208600	Psat04G0619600	Inside	Probable polygalacturonase At3g15720 like protein
3535424	4	419155845	8.34E−09	0.05	Cor21_FarmCPU_	Psat4g208600	Psat04G0619600	Inside	Probable polygalacturonase At3g15720 like protein
5879662	5	5332432	8.26E−06	7.59	Cor21_FarmCPU_	Not detected	Not detected		
5928200	5	34665431	3.73E−06	1.01	Joint_ENV_FarmCPU_	Psat5g018040	Not detected		Hyp Prot
5934000	5	56765230	1.20E−06	0.06	Cor20_BLINK_	Psat5g030080	Psat05G0119500	Inside	Oxidative stress 3 like protein
						Psat5g030120	Psat05G011960	1.23	Plant myosin class XI domain containing protein
3642481	5	81656272	6.62E−07	6.55	Cor21_BLINK_	Not detected	Not detected		
3547579	5	98406661	5.91E−06	4.05	Cor21_FarmCPU_	Psat5g054560	Psat05G0178800	Inside	Chaperone Protein ClpC
3556593	5	165347101	1.92E−06	22.56	Cor20_BLINK_	Psat5g090760	Psat05G0273300	13.67; 36.03	RNA (C5-cytosine) methyl transferase like protein
			1.18E−05	21.54	Cor21_FarmCPU_				
			1.44E−05	24.93	Joint_ENV_BLINK_				
			1.57E−07		Joint_ENV_FarmCPU_				
3546366	5	238098887	5.38E−06	24.57	Joint_ENV_FarmCPU_	Psat5g132320	Psat05G0387400	Inside	LysM-domain containing protein
5897154	5	317722532	8.15E−12	12.06	Cor21_FarmCPU_	Psat0s3165g0080	Psat05G0410900	Inside	UTP-Glucose 1-phosphate uridyltransferase like protein
			1.60E−11	13.24	Joint_ENV_FarmCPU_				
3556923	5	468430527	6.23E−07	1.1	Cor18_BLINK_	Psat5g234080	Psat05G0655000	Inside	probable LRR-receptor like protein kinase At1g53420 like protein
			2.23E−13		Cor18_FarmCPU_	Psat5g234120	Psat05G0655100	19.51; 28.31	probable LRR-receptor like protein kinase At3g14840 like protein
			2.03E−06	0.33	Cor20_BLINK_	Psat5g234040	Psat05G0654900	− 20.3; − 14.66	trichome birefringence like protein
			3.24E−06		Cor20_FarmCPU_				
5918445	5	496365311	2.12E−08	11.06	Cor19_FarmCPU_	Psat5g248280	Psat05G0698400	6.68	IAA synthase GH3.6 like protein
5885796	5	635981152	2.92E−06	8.29	Cor19_BLINK_	Psat0s2261g0080	Psat05G0818900	Inside	NRT1/PTR family 1.2 -like protein
			3.10E−06		Cor19_FarmCPU_				
			5.09E−05	10.59	Joint_ENV_BLINK_				
			4.61E−06		Joint_ENV_FarmCPU_				
3535990	5	635981632	1.86E−05	8.79	Cor21_FarmCPU_	Psat0s2261g0080	Psat05G0818900	Inside	NRT1/PTR family 1.2 -like protein
5906703	6	251955535	3.22E−06	4.29	Cor19_FarmCPU_	Psat6g131160	Psat06G0298800	Inside	SRM 1 like myb family TF
			4.87E−07	6.67	Cor20_FarmCPU_				
			2.20E−08	6.22	Joint_ENV_FarmCPU_				
5921714	6	283341356	3.58E−06	1.81	Cor21_FarmCPU_	Psat6g143280	Psat06G0386100	Inside	Serine/Threonine Protein Phosphatase PP1 like protein
3569098	6	323069753	5.12E−07	16.32	Cor21_BLINK_	Psat6g161440	Psat06G0440900	Inside	Histone-Lysine N methyltransferase domain containing protein
3557656	6	384717266	3.82E−05	3.17	Joint_ENV_BLINK_	Psat6g192360	Psat06G0517100	− 3.43; − 14.48	G-box binding factor 4-like protein (bZip TF domain)
19243729	6	399542609	8.37E−06	4.25	Joint_ENV_FarmCPU_	Psat6g201160	Psat06G0541400	Inside	E3 Ubiquitin—protein ligase Xbat33 like protein
3554365	7	47369134	3.02E−06	5.81	Cor20_FarmCPU_	Psat7g030360	Psat07G0082000	Inside	monohydroascorbate reductase like prot
5878205	7	79459335	4.21E−05	2.26	Cor21_BLINK_	Not detected	Not detected		
5953370	7	121220984	1.90E−07	1.18	Cor20_FarmCPU_	Psat7g072240	Psat07G0185000	Inside	Glucan endo-beta1-3 glucosidase 4 like protein
3564323	7	200174275	1.74E−05	2.35	Cor21_FarmCPU_	Psat7g121520	Psat07G0315500	Inside	DNA-dependent metalloprotease WSS1-like protein
3544351	7	247168420	1.46E−06	2.73	Cor21_FarmCPU_	Psat7g141960	Not detected	− 3.32	Hyp Prot
			2.45E−05	5.67	Joint_ENV_FarmCPU_	Psat7g141920	Not detected	− 25.44	BURP-domain containing Protein
3553784	7	303009045	9.09E−07	26.13	Cor21_FarmCPU_	Psat7g158440	Psat07G0514700	− 0.39; 0.79	Transport Prot SEC31 homolog B like protein
3556149	7	359047630	3.38E−06	9.03	Cor19_FarmCPU_	Psat7g190320	Psat07G0448200	− 15.52; 15.82	Stress Response A/B barrel domain containing phyp prot
19238618	Unknown		4.54E−06	8.06	Cor20_FarmCPU_				
3538403	Unknown		4.84E−09	6.1	Cor21_FarmCPU_				
			6.71E−07	8.34	Joint_ENV_FarmCPU_				
3546747	Unknown		2.42E−06	19.28	Cor21_BLINK_				
3551585	Unknown		1.52E−06	46.9	Cor19_FarmCPU_				
			1.47E−05	46.55	Joint_ENV_BLINK_				
3551680	Unknown		1.72E−05	2.99	Joint_ENV_FarmCPU_				
3558675	Unknown		3.85E−07	2.73	Joint_ENV_FarmCPU_				
8174648	Unknown		1.02E−05	7.42	Cor20_FarmCPU_				
			1.91E−07	6.99	Joint_ENV_FarmCPU_				
5935506	Scaffold0213		1.13E−05	2.13	Cor19_FarmCPU_	Psat0s213g0080	Not detected	20.84	Cytochrome P450 CYP736A12 like protein
8171869	Unknown		1.07E−06	3.24	Cor20_BLINK_				
5952035			6.20E−07	1.17	Cor18_BLINK_				
	Unknown		1.30E−06		Cor18_FarmCPU_				
			1.24E−05	2.21	Joint_ENV_BLINK_				
			1.09E−05		Joint_ENV_FarmCPU_				
3642402	Unknown		8.29E−06	5.15	Cor18_BLINK_				

These genes were annotated on the pea Caméor version1a and ZW6 reference genomes

### Identification of putative candidate genes linked to *O. crenata* resistance

**Fig. 7:**
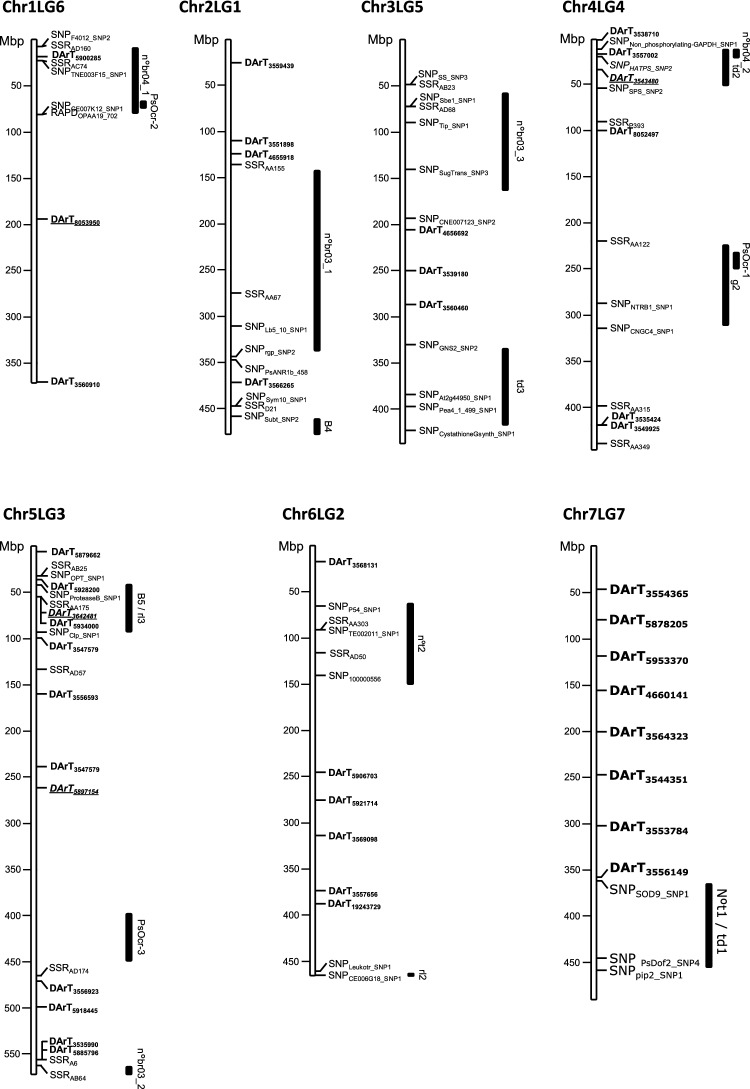
Schematic representation of the pea chromosomes showing the location of associated DArTSeq markers (in bold) and of previously identified QTL for broomrape resistance on the pea chromosomes. Location of previously identified QTLs are based on published information (Fondevilla et al. 2010; Delvento et al. 2023) with the adjacent marker positions stored in the pulse crop database (https://www.pulsedb.org/)

Putative candidate genes were identified by examining the genomic regions surrounding each MTA within two pea reference genomes (Caméor V1a and ZW6). Based on the physical position of the MTA loci, 1 to 5 annotated genes were observed within a 30 kb window for 37 of the 50 significantly associated markers. The 30 kb window size was chosen based on empirical data from the observed LD decay, and high marker density. The mean LD *r*^2^ of 0.24 placed the ideal marker distance around 30 kb. Expanding the window to 100 kb did not allow the detection of candidate genes near the 13 remaining associated markers and did not yield additional significant results. Interestingly, 24 of these markers were located in putative genes. To simplify data representation, only the closest candidate genes or more promising genes are presented in Table 5. Among them, marker 3556923 was significantly associated with broomrape resistance on chromosome 5 within the putative gene Psat5g234080. This gene encodes a member of the leucine rich repeat (LRR) receptor like kinase (RLK) family of resistance gene. Further examination of the surrounding genomic region of this marker uncovered another putative LRR-RLK gene and a trichome birefringence like protein related to defense. Two additional defense related genes (Psat04G0189200 and Psat04G0189100) were found on chromosome 4 next to marker 8052497 encoding a serine/threonine protein kinase and a plant stress related kinase (Fig. 7).

Aside plant defense genes, several genes linked to plant development and abiotic stress tolerance were identified. For instance, marker 5906703, associated with broomrape resistance in Cor19, Cor20 and Joint_ENV was included within the gene Psat6g131160 that encodes for a SMR1-like MYB transcription factor on chromosome 6. Subsequently, the marker 3543480 was found linked to gene Psat0s1953g0360 which codes for a *β*-glucosidase *sfr2*-like protein on chromosome 4. Psat1g014200 which encodes a MYB related protein was detected by marker 5900285 on chromosome 1. Marker 5934000 was found on chromosome 5 and falls within the gene, Psat5g030080 that encodes an oxidative stress 3-like protein. Similarly, markers 3556593 and 3546366 (chromosome 5) explaining more than 24% of the phenotypic variance were detected next to Psat5g090760 and Psat5g132320 that encode for an RNA methyl transferase like protein and a LysM domain containing protein respectively. In addition, two markers (5885796 and 3535990) explaining 10% of the phenotypic variance were detected within the putative gene Psat0s2261g0080 that encode a NRT1/PTR family like transporter gene on chromosome 5.

## Discussion

Pea production under Mediterranean environment is heavily affected by broomrape infection that reduces yield and quality (Fernández-Aparicio et al. 2016; Rubiales 2023). Despite longstanding efforts, no definite efficient method of control is available so far (Fernández-Aparicio et al. 2012). Genetic broomrape host resistance is the ideal control measure. Varying levels of incomplete resistance have been identified in landraces and wild relatives and introduced to advanced elite breeding lines by conventional crossing and selection methods (Rubiales et al. 2009b, 2020; Fondevilla et al. 2017). These advancements led to the development of new cultivars that are in the process of registration for future commercialization in the EU. However, this process is slow and tedious and could be significantly improved by adoption of marker assisted selection (MAS). The genetic base for resistance to crenate broomrape involves a complex inheritance, highly influenced by environmental factors (Pérez‐de‐Luque et al., 2004; Pavan et al. 2016). Several QTL associated with broomrape resistance have been identified from different bi-parental mapping populations both in pea (Valderrama et al. 2004; Fondevilla et al. 2010; Bardaro et al. 2016; Delvento et al. 2023) and in faba bean (Gutiérrez et al. 2013). However, the large distance between flanking markers and the resistance QTL impeded efficient implementation of MAS. The success of resistance breeding is dependent on wider genetic pools with a high genetic diversity and environmental adaptability. To expand the genetic basis of resistance to *O. crenata*, this study phenotyped and analyzed a genotyped pea diversity panel (Rispail et al. 2023) under field conditions. The research aimed to use GWAS to identify novel markers and candidate genes closely linked to *O. crenata* resistance.

Results of the phenotypic evaluation showed that all accessions were prone to broomrape infection, albeit infection levels varied significantly. The positive response of the pea panel exhibited a continuous distribution depicting quantitative resistance. This trend was observed by the coherent variation in the number of broomrapes per plant within and between environments. This could be indicative of the alterations in number of broomrape available seed bank at the multi-field locations. Moderate heritability was observed with infection strongly influenced by environmental factors. Previous studies in pea also demonstrated high influence of environment on broomrape development (Rubiales et al. 2014, 2020), as observed and reported in related legumes (Pérez‐de‐Luque et al., 2004; Rubiales et al. 2005; Fernández-Aparicio et al. 2011; Maalouf et al. 2011). However, the strong influence of environment was not associated with an important GEI in this research as shown by the linear models (Table 2) and GGE biplot (Fig. 3). The environment average (TEA) departed only slightly from the biplot origin supporting the relatively low GEI (Fig. 2). The accessions closest to the origin were less sensitive to environmental variation, while the farthest were more responsive. Accordingly, accessions #317, #308, #311, #276, and #245 that were farther away from the origin and opposite to the environment vectors were the most resistant to broomrape. On the other hand, accessions #19, #55, and #114 that are in the same direction as the environment vectors were the most susceptible. Differential performance may be attributed to variation in yearly rainfall and prevailing environmental derivatives (Table 1). This attribute was also observed in faba bean fields, demonstrating that the phenotypic expression of broomrape emergence is unstable across environments (Rubiales et al. 2014). Thus, trait-environment linkages provides a priori information that can enhance FIGS (focused identification germplasm strategy) to guide the efficient selection of accessions for stress adaptation in legumes (Khazaei et al. 2013; Bohra et al. 2024). FIGS approach augments the efficient targeting and utilization of promising accessions with specific traits, which promote the discovery of novel alleles under selective environmental pressure.

Wild pea accessions from the Middle East within the *P. fulvum*, *P. abyssinicum*, and *P. sativum* subsp*. elatius* var. *elatius* showed higher broomrape resistance than the accessions of *P. sativum* subsp. *sativum* var. *sativum, P. sativum* subsp. *sativum* var. *arvense* and *P. sativum* subsp. *jomardii*. This confirms the potential of wild relatives as resistance gene reservoirs for breeding (Rubiales et al. 2005; Smýkal et al. 2017; Coyne et al. 2020). Interestingly, this zone is considered a center of pea origin, suggesting the potential for local adaptation and the evolution of broomrape-host interactions within this region. Further in-depth investigation could determine the geographic origins of resistance and susceptibility in these accessions to enhance targeted breeding strategies. Despite relatively narrow GEI detected in the pea panel, the linear mixed model deployed accurately predicted the quantitative response of broomrape infection in pea and identified novel sources of partial resistance. Phenotypic results revealed promising candidate accessions that could be exploited for resistance breeding (Table 3), but integrating these results with genomic data are more informative and elucidates genomic regions linked to *O. crenata* resistance through GWAS.

With the availability of novel markers and cost-effective genotyping approaches, GWAS based on the non-random linkage of loci can optimize high-resolution mapping of quantitative traits to accelerate breeding (Myles and Wayne 2008). GWAS was deployed to significantly enhanced the predictive capabilities of genomic models in a pea collection (Zhang et al. 2019; Crosta et al. 2023). Thus, selection of quality robust markers and ideal germplasm size can enhance breeding efficiency. Genetic analysis of this collection revealed extensive level of LD between markers, with a high density of markers (0.166 Mbp average spacing) (Rispail et al. 2023). This high chromosomal level marker coverage is considered efficient and optimal for delineating QTL in association mapping. Since population structure can greatly affect association study outcomes, the structure of the pea diversity panel was examined with ADMIXTURE prior to GWAS. This indicated the presence of 4 to 6 subpopulation in the collection (Fig. 4) confirming previous STRUCTURE and PCA analysis of the diversity panel (Rispail et al. 2023). This also confirmed the highly admixed nature of most landraces from *P. sativum* subsp. *sativum* var. *arvense* and *P. sativum* subsp. *jomardii* (Rispail et al. 2023). In the present GWAS, 26,045 quality Silico-DArT markers and FarmCPU/BLINK models were implemented in GAPIT. GWAS elucidated the genetic architecture of partial broomrape resistance in this pea collection. Confounding effects were mitigated using kinship and PCs to control for population structure. QQ plots verification revealed that, the models were adequately calibrated and confirmed the significant hits captured by the Manhattan plots. GWAS identified 73 significant MTAs with broomrape resistance in the field, highlighting the complexity of broomrape resistance. Associated markers were detected on all seven pea chromosomes, showcasing the robustness and usefulness of DarT markers in trait discovery. In addition, most MTAs explained a small portion of the phenotypic variance confirming the complexity and low heritability of broomrape resistance previously described in pea (Rispail et al. 2007; Rubiales et al. 2009b). Interestingly, five markers explained more than 20% of the phenotypic variance each, which offer an unprecedented opportunity for enhancing broomrape resistance level through MAS. Chromosome 5 presented the highest hotspot of significant MTAs with 22 associations, including 12 SilicoDArT markers, while chromosome 3 had only two MTAs. Significant MTAs corresponding to markers that could not be mapped onto any pea reference genomes may be within noncoding regions, thus future whole genome sequencing may hold the key to unraveling such traits (Uffelmann et al. 2021). FarmCPU model exhibited greater robustness by iteratively applying fixed and random effect models, while BLINK employed a BIC-based approach that, although computationally powerful, might have compromised efficiency (Wang and Zhang 2021). This was in agreement with recent association studies in chickpea using the same models, where FarmCPU outperformed the BLINK model in controlling *p-value* inflation and finding linked markers (Kumar et al. 2025). However, both models were retained in this studies to address the limitations of single-locus models, maximize the identification of MTAs, and optimize locus discovery (Li et al. 2018). Mapping the significantly associated markers onto the pea genetic map revealed that some of these markers fall within the confidence intervals of previously identified QTLs (*nºbr03_2, nºbr04_1, nºbr04_2, Td2,* and *B5*) associated with broomrape resistance (Fondevilla et al. 2010). This highlights the effectiveness of GWAS and allows refining these previously described QTL. In addition, these associated markers could be useful as a foundation for improving *O. crenata* resistance level in cultivars. None of the associated markers co-localized with the recently described QTL, *PsOcr-1*, *PsOcr-2* and *PsOcr-3* that were detected from another bi-parental mapping population, although some associated SilicoDArT markers fall in close vicinity to these loci (Delvento et al. 2023).

The significant MTAs were located on the Caméor and ZW6 reference genomes at a 30 kb window to identify putative genes. Out of the 50 significantly associated markers, 1 to 5 annotated genes were found within the 30 kb window of 37 markers with 24 of these markers located within a predicted gene. This confirms the efficiency of DArT markers suggested to be linked to gene rich regions (Barilli et al. 2018), thus facilitating the identification of candidate genes. Further annotation and literature review revealed that the candidate genes might involve several functions relating to pea growth, development indices and stress responses. The candidate genes were mainly participating in cell signaling and trafficking, regulation of transcription, metabolite transporter, primary and secondary metabolism, and defense. Interestingly, the marker 3556923, was located within the gene, Psat5g234080 that encodes a leucine rich repeat (LRR) receptor like kinase (RLK) family of resistance genes on chromosome 5. Likewise, marker (3556923) was also in significant proximity to a previous QTL (*PsOcr-3*) elucidated by Delvento et al. (2023). On the same locus, another LRR-RLK gene and a trichome birefringence-like protein was found associated with defense reaction in Arabidopsis (Kulich et al. 2015). LRR-RLK family protein was found to contain a binding site for small signaling peptide and phytosulfokine signaling to promotes normal growth and development and also plays a role in defense responses (Nagar et al. 2020). We identified defense-related genes near marker 8052497 on chromosome 4, associated to a serine/threonine protein kinase and a plant stress-related kinase (Psat4g059880/ Psat04G0189200). This marker was found in the zone of *PsOcr-1* locus (Delvento et al. 2023). The gene (Psat6g131160) coding for a SMR1-like Myb transcription factor was identified by marker 5906703 on chromosome 6 associated with broomrape resistance in different environments. This could be ideal for selecting broomrape resistance in multiple environments. Similarly, marker 5900285 detected a Myb transcription related protein, Psat1g014200 within the confidence interval of the previously identified QTL, *nºbr04_1* on chromosome 1. Most recently, this Myb-like transcription factor was elucidated to be involved in regulating secondary metabolism, such as the biosynthesis of flavonoids and other phenolic compounds which contribute to plant defense and pigmentation (Li et al. 2023). Furthermore, marker 3543480 was found linked to gene Psat0s1953g0360 which codes for a *β*-glucosidase *sfr2*-like protein on chromosome 4. This gene was within the confidence interval of a previous QTL (*td2*) associated with broomrape tubercule development under controlled rhizotron studies (Fondevilla et al. 2010). In common bean, *β*-glucosidase exhibited the highest increase in abundance among proteins involved in cell wall metabolism (Zadražnik et al. 2017). Accordingly, it may play a role in cell wall strengthening to maintain the cell wall integrity of pea to prevent early-stage attachment of broomrape tubercles. Cell wall fortification encoding genes that uses lignin deposition biosynthesis were also found to provoke *Striga hermontica* resistance in sorghum (Kavuluko et al. 2021). In addition, marker 5934000 located on chromosome 5 was located within the coding region of Psat5g030080 that encodes an oxidative stress 3-like protein found to regulate abiotic stress in legumes (Sharma et al. 2021). Markers 3556593 and 3546366 (chromosome 5) were detected next to Psat5g090760 and Psat5g132320 that encodes for an RNA methyl transferase-like protein and a LysM domain containing protein. This LysM-domain containing proteins are noted to be receptors of fungal elicitors that induces defense in *A. thaliana* (Nakagawa et al. 2011). Finally, two markers (5885796 and 3535990) were detected within the putative gene Psat0s2261g0080 that encode a NRT1/PTR family like transporter gene. NRT1/PTR family genes play a crucial role in plant development, serving as both a nutrient transporter and a signaling molecule. NRT1/PTR genes are also responsible for nitrate transport in legumes and abscisic acid, auxin, dipeptides, and glycosylates signaling transduction and plant development (Léran et al. 2014; Pellizzaro et al. 2017). These markers elaborated here are expected to improve the understanding of genetic loci governing broomrape resistance.

Conclusively, to better understand pea resistance to broomrape, there is the need to study broomrape virulence and pea resistance mechanisms. Developing in vitro protocols for pea-broomrape interactions can improve phenotypic evaluation and QTL detection (Fondevilla et al. 2010; Fernández-Aparicio et al. 2012; Wohor et al. 2022). Marker-trait association is a fundamental requirement for MAS, which enables the identification of target traits without the need for phenotypic evaluation in early generations. Thus, this study has successfully validated 4 previous broomrape resistance QTLs and efficiently used DArT markers to identify novel MTAs for elucidating candidate genes that participated in broomrape resistance under field conditions. Chromosome 5, and 4 appear to be the hotspot for genes related to defense and stress genes linked to broomrape resistance (Fig. 4). Discovery of these markers linked to broomrape resistance are promising, as current management options are limited—existing methods are often either economically impractical or harmful to the environment (Rubiales and Fernández-Aparicio 2012; Rubiales 2023). Moreover, most research efforts against pea broomrapes has only yielded incomplete level of resistance (Rubiales et al. 2009b). Therefore, the identified genes have the potential for marker-assisted breeding if further enhanced through functional studies, leading to the introgression of found loci into breeding programs to facilitate the development of broomrape resistant pea cultivars.

## Supplementary Information

Below is the link to the electronic supplementary material.

Supplementary file1 (DOCX 52 KB)

## Data Availability

The DArTseq marker data used for the current study are available in the Zenodo repository, https://zenodo.org/records/7180467. Phenotypic data of the study are available as supplementary files and available from the authors on reasonable request.
