# Supplementary material for: Genome wide association study unveils the genetic basis of Orobanche crenata resistance in pea
Source: Theor Appl Genet. 2025 Oct 11;138(11):272. doi: 10.1007/s00122-025-05051-2 (PMC12515232; doi:10.1007/s00122-025-05051-2)
Supplement: Supplementary file 1 — Supplementary file1 (DOCX 52 KB) [file 122_2025_5051_MOESM1_ESM.docx]

**Supplementary Table 1.** List of accessions composing the pea diversity panel and their phenotypic response to broomrape under field condition.

| **Acc nº** | **Bank Code** | **Species / subspecies** | **Origin** | **Cor18** | **Cor19** | **Cor20** | **Cor21** | **Joint_ENV** |
| --- | --- | --- | --- | --- | --- | --- | --- | --- |
| 1 | PI 109865 | *P. sativum* subsp. *sativum* var. *arvense* | Venezuela | 1.88 | 3.62 | 2.61 | 3.68 | 2.95 |
| 2 | PI 117910 | *P. sativum* subsp. *sativum* var.*sativum* | Brazil | 1.48 | 2.48 | 1.95 | 2.85 | 2.19 |
| 3 | PI 140297 | *P. sativum* subsp. *sativum* var. *sativum* | Iran | 2.17 | 1.60 | 1.71 | 1.87 | 1.84 |
| 4 | PI 142442 | *P. sativum* subsp. *sativum* var. *arvense* | Peru | 1.78 | 1.67 | 1.11 | 2.38 | 1.74 |
| 5 | PI 142774 | *P. sativum* subsp. *jomardii* | Mexico | 3.14 | 2.32 | 0.59 | 4.66 | 2.68 |
| 6 | PI 142776 | *P. sativum* subsp. *sativum* var. *arvense* | Mexico | 2.05 | 2.22 | 2.17 | 2.30 | 2.18 |
| 7 | PI 142776 | *P. sativum* subsp. *sativum* var. *arvense* | Mexico | 2.29 | 2.88 | 2.36 | 1.94 | 2.37 |
| 8 | PI 143483 | *P. sativum* subsp. *sativum* var. *sativum* | Azerbaijan | 5.65 | 1.85 | 4.67 | 3.40 | 3.89 |
| 9 | PI 143484 | *P. sativum* subsp. *jomardii* | Azerbaijan | 3.82 | 2.13 | 1.58 | 4.22 | 2.94 |
| 10 | PI 143486 | *P. sativum* subsp. *sativum* var. *sativum* | Iran | 1.96 | 1.40 | 0.55 | 2.10 | 1.50 |
| 11 | PI 153351 | *P. sativum* subsp. *sativum* var. *arvense* | Ecuador | 1.87 | 3.02 | 1.36 | 2.55 | 2.20 |
| 12 | PI 162568 | *P. sativum* subsp. *sativum* var. *sativum* | Argentina | 2.27 | 4.55 | 2.16 | 2.64 | 2.90 |
| 13 | PI 162692 | *P. sativum* subsp. *jomardii* | Argentina | 1.83 | 2.26 | 1.47 | 2.84 | 2.10 |
| 14 | PI 162693 | *P. sativum* subsp. *sativum* var. *arvense* | Argentina | 3.74 | 2.72 | 1.04 | 3.18 | 2.67 |
| 15 | PI 162693 | *P. sativum* subsp. *sativum* var. *arvense* | Argentina | 3.00 | 2.32 | 3.65 | 3.31 | 3.07 |
| 16 | PI 162910 | *P. sativum* subsp. *sativum* var. *sativum* | Paraguay | 0.81 | 1.74 | 1.78 | 1.92 | 1.56 |
| 17 | PI 164568 | *P. sativum* subsp. *sativum* var. *arvense* | India | 2.63 | 3.24 | 2.33 | 3.00 | 2.80 |
| 18 | PI 166082 | *P. sativum* subsp. *elatius* var. *pumilio* | India | 1.37 | 1.07 | 2.00 | 1.27 | 1.43 |
| 19 | PI 195405 | *P. sativum* subsp. *sativum* var. *arvense* | Guatemala | 1.07 | 1.75 | 2.41 | 3.22 | 2.11 |
| 20 | PI 203065 | *P. sativum* subsp. *jomardii* | Finland | 3.00 | 0.92 | 1.99 | 3.84 | 2.44 |
| 21 | PI 204305 | *P. sativum* subsp. *sativum* var. *sativum* | Australia | 0.14 | 0.11 | 0.09 | 0.46 | 0.20 |
| 22 | PI 204667 | *P. sativum* subsp. *sativum* var. *sativum* | Netherland | 0.63 | 1.55 | 1.47 | 1.49 | 1.28 |
| 23 | PI 220175 | *P. sativum* subsp. *elatius* var. *pumilio* | Afganistan | 0.07 | 1.75 | 0.14 | 0.79 | 0.69 |
| 24 | PI 220673 | *P. sativum* subsp. *sativum* var. *arvense* | Afganistan | 1.68 | 1.45 | 1.77 | 2.29 | 1.80 |
| 25 | PI 222069 | *P. sativum* subsp. *elatius* var. *pumilio* | Afganistan | 0.72 | 0.78 | 1.37 | 0.44 | 0.83 |
| 26 | PI 234262 | *P. sativum* subsp. *sativum* var. *sativum* | USA | 1.84 | 0.85 | 1.06 | 1.68 | 1.36 |
| 27 | PI 254625 | *P. sativum* subsp. *jomardii* | Finland | 0.98 | 2.14 | 2.38 | 3.53 | 2.26 |
| 28 | PI 254626 | *P. sativum* subsp. *jomardii* | Australia | 1.31 | 2.82 | 1.01 | 2.75 | 1.97 |
| 29 | PI 261678 | *P. sativum* subsp. *jomardii* | Netherland | 3.22 | 3.03 | 5.39 | 4.36 | 4.00 |
| 30 | PI 262189 | *P. sativum* subsp. *sativum* var. *arvense* | Costa Rica | 1.19 | 3.08 | 2.36 | 3.44 | 2.52 |
| 31 | PI 266069 | *P. sativum* subsp. *sativum* var. *arvense* | Sweden | 1.06 | 2.70 | 1.09 | 2.97 | 1.95 |
| 32 | PI 269760 | *P. sativum* subsp. *sativum* var. *arvense* | UK | 3.03 | 2.86 | 3.86 | 4.35 | 3.53 |
| 33 | PI 269763 | *P. sativum* subsp. *jomardii* | UK | 2.01 | 2.58 | 4.45 | 2.57 | 2.90 |
| 34 | PI 269786 | *P. sativum* subsp. *sativum* var. *sativum* | UK | 1.02 | 0.87 | 1.48 | 2.38 | 1.44 |
| 35 | PI 272143 | *P. sativum* subsp. *jomardii* | Germany | 3.67 | 3.43 | 1.93 | 4.08 | 3.28 |
| 36 | PI 272151 | *P. sativum* subsp. *sativum* var. *arvense* | Germany | 1.68 | 2.77 | 1.39 | 2.09 | 1.98 |
| 37 | PI 272153 | *P. sativum* subsp. *jomardii* | Greece | 2.01 | 1.73 | 1.88 | 2.57 | 2.05 |
| 38 | PI 272156 | *P. sativum* subsp. *jomardii* | Greece | 1.56 | 1.45 | 0.93 | 1.81 | 1.44 |
| 39 | PI 280621 | *P. sativum* subsp. *sativum* var. *sativum* | Rusia | 0.43 | 1.24 | 0.70 | 2.70 | 1.27 |
| 40 | PI 280623 | *P. sativum* subsp. *jomardii* | Poland | 2.18 | 4.49 | 1.65 | 2.28 | 2.65 |
| 41 | PI 306592 | *P. sativum* subsp. *jomardii* | Hungary | 1.12 | 2.48 | 1.14 | 2.92 | 1.91 |
| 42 | PI 312136 | *P. sativum* subsp. *sativum* var. *arvense* | Guatemala | 3.97 | 2.95 | 5.29 | 3.60 | 3.95 |
| 43 | PI 319373 | *P. sativum* subsp. *sativum* var. *arvense* | Mexico | 1.08 | 1.20 | 4.42 | 4.04 | 2.69 |
| 44 | PI 326194 | *P. sativum* subsp. *jomardii* | Mexico | 1.54 | 1.97 | 3.12 | 1.95 | 2.15 |
| 45 | PI 343326 | *P. sativum* subsp. *sativum “indian ecotype”* | USA | 1.40 | 0.97 | 3.14 | 3.72 | 2.31 |
| 46 | PI 343329 | *P. sativum* subsp. *sativum* var. *sativum* | USA | 1.80 | 1.86 | 0.81 | 2.68 | 1.79 |
| 47 | PI 343935 | *P. sativum* subsp. *sativum* var. *arvense* | Ethiopia | 2.38 | 2.06 | 1.99 | 2.27 | 2.17 |
| 48 | PI 343962 | *P. sativum* subsp. *sativum* var. *arvense* | Turkey | 0.58 | 3.37 | 0.82 | 1.58 | 1.59 |
| 49 | PI 343965 | *P. sativum* subsp. *sativum* var. *arvense* | Turkey | 1.91 | 1.86 | 2.19 | 3.18 | 2.28 |
| 50 | PI 343965 | *P. sativum* subsp. *jomardii* | Turkey | 0.97 | 1.54 | 3.59 | 4.35 | 2.61 |
| 51 | PI 343969 | *P. sativum* subsp. *sativum* var. *sativum* | Turkey | 0.73 | 1.89 | 1.30 | 3.03 | 1.74 |
| 52 | PI 343981 | *P. sativum* subsp. *sativum* var. *sativum* | Turkey | 0.26 | 1.25 | 0.86 | 2.60 | 1.24 |
| 53 | PI 343984 | *P. sativum* subsp. *sativum* var. *arvense* | Turkey | 3.12 | 1.25 | 1.97 | 3.23 | 2.39 |
| 54 | PI 343993 | *P. sativum* subsp. *jomardii* | Turkey | 0.00 | 0.00 | 0.00 | 0.19 | 0.05 |
| 55 | PI 347282 | *P. sativum* subsp. *sativum “indian ecotype”* | India | 5.03 | 0.70 | 4.35 | 3.32 | 3.35 |
| 56 | PI 347316 | *P. sativum* subsp. *sativum* var. *arvense* | India | 2.77 | 3.00 | 0.61 | 3.42 | 2.45 |
| 57 | PI 347317 | *P. sativum* subsp. *sativum “indian ecotype”* | India | 1.34 | 3.39 | 1.73 | 3.53 | 2.50 |
| 58 | PI 347319 | *P. sativum* subsp. *sativum* var. *arvense* | India | 1.85 | 2.22 | 2.12 | 2.90 | 2.27 |
| 59 | PI 347321 | *P. sativum* subsp. *sativum “indian ecotype”* | India | 1.18 | 1.73 | 2.68 | 4.59 | 2.55 |
| 60 | PI 347323 | *P. sativum* subsp. *sativum “indian ecotype”* | India | 1.46 | 2.03 | 2.20 | 2.34 | 2.01 |
| 61 | PI 347326 | *P. sativum* subsp. *sativum “indian ecotype”* | India | 0.82 | 3.74 | 1.26 | 2.49 | 2.08 |
| 62 | PI 347328 | *P. sativum* subsp. *sativum* var. *arvense* | India | 2.88 | 2.55 | 2.93 | 2.01 | 2.59 |
| 63 | PI 347330 | *P. sativum* subsp. *sativum “indian ecotype”* | India | 0.41 | 1.73 | 2.46 | 3.94 | 2.13 |
| 64 | PI 347332 | *P. sativum* subsp. *sativum “indian ecotype”* | India | 2.00 | 3.70 | 1.62 | 1.87 | 2.30 |
| 65 | PI 347333 | *P. sativum* subsp. *sativum “indian ecotype”* | India | 1.61 | 2.23 | 2.46 | 1.90 | 2.05 |
| 66 | PI 347334 | *P. sativum* subsp. *sativum “indian ecotype”* | India | 1.40 | 2.54 | 3.15 | 3.25 | 2.58 |
| 67 | PI 347335 | *P. sativum* subsp. *sativum “indian ecotype”* | India | 1.59 | 2.28 | 3.09 | 1.87 | 2.21 |
| 68 | PI 347336 | *P. sativum* subsp. *sativum “indian ecotype”* | India | 3.02 | 1.72 | 1.70 | 3.56 | 2.50 |
| 69 | PI 347338 | *P. sativum* subsp. *sativum* var. *arvense* | India | 0.71 | 1.89 | 0.26 | 1.89 | 1.19 |
| 70 | PI 347342 | *P. sativum* subsp. *sativum* var. *arvense* | India | 0.32 | 1.32 | 0.91 | 3.71 | 1.56 |
| 71 | PI 347343 | *P. sativum* subsp. *sativum* var. *arvense* | India | 1.44 | 1.61 | 3.45 | 5.07 | 2.89 |
| 72 | PI 347347 | *P. sativum* subsp. *sativum “indian ecotype”* | India | 4.06 | 1.47 | 2.35 | 3.63 | 2.88 |
| 73 | PI 347348 | *P. sativum* subsp. *sativum “indian ecotype”* | India | 3.33 | 0.96 | 2.94 | 3.41 | 2.66 |
| 74 | PI 347356 | *P. sativum* subsp. *sativum “indian ecotype”* | India | 2.25 | 2.70 | 1.54 | 2.52 | 2.25 |
| 75 | PI 347357 | *P. sativum* subsp. *sativum “indian ecotype”* | India | 1.00 | 1.84 | 1.99 | 2.64 | 1.87 |
| 76 | PI 347359 | *P. sativum* subsp. *sativum “indian ecotype”* | India | 1.08 | 1.08 | 2.63 | 1.90 | 1.67 |
| 77 | PI 347366 | *P. sativum* subsp. *sativum* var. *arvense* | India | 0.29 | 1.33 | 1.17 | 1.37 | 1.04 |
| 78 | PI 347367 | *P. sativum* subsp. *sativum “indian ecotype”* | India | 2.80 | 1.45 | 2.89 | 3.55 | 2.67 |
| 79 | PI 347370 | *P. sativum* subsp. *sativum “indian ecotype”* | India | 2.19 | 2.67 | 1.67 | 2.48 | 2.25 |
| 80 | PI 347372 | *P. sativum* subsp. *sativum* var. *arvense* | India | 1.07 | 1.55 | 2.06 | 2.52 | 1.80 |
| 81 | PI 347373 | *P. sativum* subsp. *sativum* var. *arvense* | India | 0.97 | 0.99 | 1.84 | 1.68 | 1.37 |
| 82 | PI 347374 | *P. sativum* subsp. *sativum* var. *arvense* | India | 0.33 | 1.39 | 0.82 | 2.36 | 1.23 |
| 83 | PI 347375 | *P. sativum* subsp. *sativum* var. *arvense* | India | 2.51 | 2.88 | 1.90 | 3.79 | 2.77 |
| 84 | PI 347383 | *P. sativum* subsp. *sativum* var. *arvense* | India | 0.96 | 1.20 | 1.97 | 3.51 | 1.91 |
| 85 | PI 347385 | *P. sativum* subsp. *sativum “indian ecotype”* | India | 1.29 | 2.73 | 2.72 | 2.83 | 2.39 |
| 86 | PI 347388 | *P. sativum* subsp. *sativum* var. *arvense* | India | 1.64 | 2.95 | 1.80 | 3.22 | 2.40 |
| 87 | PI 347389 | *P. sativum* subsp. *sativum* var. *arvense* | India | 0.09 | 1.60 | 0.65 | 0.84 | 0.79 |
| 88 | PI 347401 | *P. sativum* subsp. *sativum “indian ecotype”* | India | 0.23 | 2.14 | 2.63 | 2.13 | 1.78 |
| 89 | PI 347471 | *P. sativum* subsp. *sativum* var. *sativum* | India | 0.68 | 2.03 | 1.04 | 3.80 | 1.89 |
| 90 | PI 358642 | *P. sativum* subsp. *sativum* var. *sativum* | Ethiopia | 0.72 | 1.57 | 1.19 | 3.08 | 1.64 |
| 91 | PI 379612 | *P. sativum* subsp. *jomardii* | Sweden | 3.51 | 2.88 | 4.97 | 5.52 | 4.22 |
| 92 | PI 385981 | *P. sativum* subsp. *sativum* var. *sativum* | UK | 1.24 | 2.60 | 2.50 | 2.20 | 2.14 |
| 93 | PI 399129 | *P. sativum* subsp. *sativum* var. *sativum* | Germany | 0.79 | 0.70 | 0.24 | 0.51 | 0.56 |
| 94 | PI 494079 | *P. sativum* subsp. *sativum* var. *sativum* | Chile | 1.96 | 4.84 | 2.30 | 3.30 | 3.10 |
| 95 | PI 560065 | *P. fulvum* | Israel | 0.00 | 0.00 | 0.05 | 0.10 | 0.04 |
| 96 | PI 560067 | *P. fulvum* | Israel | 0.00 | 0.07 | 0.30 | 0.00 | 0.09 |
| 97 | PI 595933 | *P. fulvum* | Australia | 0.00 | 0.00 | 0.21 | 0.27 | 0.12 |
| 98 | PI 595945 | *P. fulvum* | Jordan | 0.00 | 0.03 | 0.66 | 0.35 | 0.26 |
| 99 | PI 595947 | *P. fulvum* | Israel | 0.08 | 0.30 | 0.13 | 0.25 | 0.19 |
| 100 | JI 85 | *P. sativum* subsp. *elatius* var. *pumilio* | Afganistan | 0.70 | 1.23 | 2.22 | 1.33 | 1.37 |
| 101 | JI 156 | *P. sativum* subsp. *jomardii* | Sudan | 0.00 | 0.17 | 1.39 | 0.43 | 0.50 |
| 102 | JI 156 | *P. sativum* subsp. *jomardii* | Sudan | 0.54 | 0.97 | 0.99 | 0.75 | 0.81 |
| 103 | JI 262 | *P. sativum* subsp. *elatius* var. *elatius* | Turkey | 0.07 | 0.28 | 0.30 | 0.17 | 0.21 |
| 104 | JI 263 | *P. sativum* subsp. *jomardii* | Greece | 0.08 | 0.58 | 0.56 | 0.86 | 0.52 |
| 105 | JI 228 | *P. sativum* subsp. *sativum* var. *arvense* | Bolivia | 1.20 | 1.58 | 1.61 | 2.24 | 1.66 |
| 106 | JI 209 | *P. sativum* subsp. *jomardii* | India | 0.08 | 0.44 | 0.39 | 0.10 | 0.25 |
| 107 | JI 209 | *P. sativum* subsp. *sativum* var. *arvense* | India | 0.56 | 0.64 | 1.07 | 2.32 | 1.15 |
| 108 | JI 207 | *P. sativum* subsp. *jomardii* | UZBEKISTAN | 2.10 | 1.64 | 2.11 | 3.03 | 2.22 |
| 109 | JI 224 | *P. fulvum* | Israel | 0.00 | 0.03 | 0.00 | 0.09 | 0.03 |
| 110 | JI 196 | *P. sativum* subsp. *jomardii* | Georgia | 0.94 | 1.32 | 1.11 | 1.98 | 1.34 |
| 111 | JI 190 | *P. sativum* subsp. *jomardii* | Sudan | 0.36 | 0.35 | 1.31 | 1.20 | 0.80 |
| 112 | JI 189 | *P. sativum* subsp. *jomardii* | Sudan | 0.05 | 0.85 | 0.38 | 0.42 | 0.42 |
| 113 | JI 185 | *P. sativum* subsp. *jomardii* | Sudan | 0.01 | 0.09 | 0.04 | 0.18 | 0.08 |
| 114 | JI 267 | *P. sativum* subsp. *jomardii* | Greece | 0.32 | 5.48 | 1.62 | 2.29 | 2.43 |
| 115 | JI 268 | *P. sativum* subsp. *jomardii* | Crete | 0.42 | 0.03 | 0.19 | 0.36 | 0.25 |
| 116 | JI 275 | *P. sativum* subsp. *sativum* var. *arvense* | Crete | 2.11 | 2.14 | 3.23 | 2.01 | 2.37 |
| 117 | JI 280 | *P. sativum* subsp. *jomardii* | Albania | 2.36 | 1.70 | 2.62 | 2.49 | 2.30 |
| 118 | JI 288 | *P. sativum* subsp. *sativum* var. *arvense* | Greece | 1.72 | 2.80 | 1.72 | 1.09 | 1.83 |
| 119 | JI 502 | *P. sativum* subsp. *sativum* var. *sativum* | Netherland | 0.64 | 1.43 | 1.41 | 2.02 | 1.37 |
| 120 | JI 701 | *P. sativum* subsp. *sativum* var. *arvense* | Italy | 1.00 | 3.23 | 1.64 | 3.24 | 2.28 |
| 121 | JI 1030 | *P. sativum* subsp. *sativum* var. *arvense* | Iran | 1.47 | 2.23 | 1.34 | 2.92 | 1.99 |
| 122 | JI 1057 | *P. sativum* subsp. *sativum* var. *arvense* | Colombia | 0.24 | 0.73 | 0.57 | 0.54 | 0.52 |
| 123 | JI 1089 | *P. sativum* subsp. *jomardii* | Turkey | 0.15 | 0.71 | 1.66 | 0.67 | 0.80 |
| 124 | JI 1107 | *P. sativum* subsp. *elatius* var. *pumilio* | Nepal | 1.02 | 0.95 | 1.25 | 0.80 | 1.01 |
| 125 | JI 1213 | *P. sativum* subsp. *sativum* var. *sativum* | France | 2.66 | 1.82 | 1.02 | 1.09 | 1.64 |
| 126 | JI 1345 | *P. sativum* subsp. *jomardii* | Mongolia | 2.62 | 3.45 | 1.49 | 2.00 | 2.39 |
| 127 | JI 1346 | *P. sativum* subsp. *elatius* var. *pumilio* | Mongolia | 1.41 | 2.03 | 2.57 | 1.50 | 1.88 |
| 128 | JI 2263 | *P. sativum* subsp. *jomardii* | Germany | 0.37 | 0.34 | 2.12 | 3.10 | 1.48 |
| 129 | JI 2265 | *P. sativum* subsp. *jomardii* | Albania | 1.62 | 2.34 | 0.98 | 1.96 | 1.72 |
| 130 | JI 2356 | *P. sativum* subsp. *sativum* var. *arvense* | Nepal | 0.19 | 0.66 | 0.25 | 0.64 | 0.44 |
| 131 | JI 2385 | *P. abyssinicum* | Yemen | 0.25 | 0.35 | 0.56 | 1.05 | 0.55 |
| 132 | JI 2387 | *P. sativum* subsp. *sativum* var. *arvense* | Ethiopia | 0.70 | 0.62 | 1.60 | 1.63 | 1.14 |
| 133 | JI 2545 | *P. sativum* subsp. *elatius* var. *pumilio* | Pakistan | 1.93 | 1.12 | 3.17 | 1.81 | 2.01 |
| 134 | BGE001004 | *P. sativum* subsp. *jomardii* | Spain | 2.29 | 2.41 | 2.83 | 2.54 | 2.52 |
| 135 | BGE001034 | *P. sativum* subsp. *sativum* var. *arvense* | Spain | 1.62 | 1.69 | 1.23 | 1.68 | 1.56 |
| 136 | BGE001121 | *P. sativum* subsp. *sativum* var. *arvense* | Spain | 0.34 | 3.02 | 0.62 | 2.83 | 1.70 |
| 137 | BGE001121 | *P. sativum* subsp. *sativum* var. *arvense* | Spain | 0.65 | 1.44 | 0.50 | 0.78 | 0.84 |
| 138 | BGE001662 | *P. sativum* subsp. *jomardii* | Spain | 1.84 | 0.88 | 2.01 | 2.30 | 1.76 |
| 139 | BGE002168 | *P. sativum* subsp. *jomardii* | Spain | 1.52 | 3.51 | 3.68 | 1.84 | 2.64 |
| 140 | BGE002168 | *P. sativum* subsp. *jomardii* | Spain | 3.15 | 2.65 | 4.22 | 4.02 | 3.51 |
| 141 | BGE003315 | *P. sativum* subsp. *sativum* var. *arvense* | Spain | 2.35 | 3.08 | 2.78 | 4.88 | 3.27 |
| 142 | BGE004710 | *P. sativum* subsp. *sativum* var. *arvense* | Portugal | 2.40 | 3.11 | 2.06 | 2.99 | 2.64 |
| 143 | BGE004713 | *P. sativum* subsp. *jomardii* | Portugal | 3.01 | 3.71 | 5.73 | 3.50 | 3.99 |
| 144 | BGE004958 | *P. sativum* subsp. *jomardii* | Portugal | 2.90 | 3.19 | 5.63 | 2.77 | 3.63 |
| 145 | BGE006125 | *P. sativum* subsp. *sativum* var. *arvense* | Portugal | 3.66 | 3.88 | 4.02 | 6.98 | 4.64 |
| 146 | BGE006126 | *P. sativum* subsp. *sativum* var. *sativum* | Portugal | 1.72 | 1.56 | 1.44 | 2.29 | 1.75 |
| 147 | BGE019594 | *P. sativum* subsp. *sativum* var. *arvense* | Spain | 1.02 | 1.47 | 0.83 | 1.18 | 1.13 |
| 148 | BGE022159 | *P. sativum* subsp. *jomardii* | Spain | 3.50 | 2.56 | 3.19 | 7.47 | 4.18 |
| 149 | BGE020326 | *P. sativum* subsp. *sativum* var. *arvense* | Spain | 4.56 | 4.69 | 8.77 | 4.86 | 5.72 |
| 150 | BGE023256 | *P. sativum* subsp. *jomardii* | Spain | 1.78 | 3.19 | 1.53 | 3.58 | 2.52 |
| 151 | BGE025263 | *P. sativum* subsp. *sativum* var. *sativum* | Spain | 2.97 | 6.40 | 4.33 | 2.97 | 4.17 |
| 152 | BGE025267 | *P. sativum* subsp. *jomardii* | Spain | 1.54 | 1.94 | 2.15 | 1.91 | 1.89 |
| 153 | BGE025270 | *P. sativum* subsp. *jomardii* | Spain | 2.99 | 2.46 | 1.20 | 2.87 | 2.38 |
| 154 | BGE026428 | *P. sativum* subsp. *sativum* var. *sativum* | Spain | 0.74 | 1.19 | 1.17 | 0.86 | 0.99 |
| 155 | BGE026429 | *P. sativum* subsp. *sativum* var. *arvense* | Spain | 3.89 | 4.52 | 3.66 | 6.56 | 4.66 |
| 156 | CGN16690 | *P. sativum* subsp. *jomardii* | Italy | 1.67 | 2.08 | 4.03 | 3.65 | 2.86 |
| 157 | CGN03277 | *P. sativum* subsp. *elatius* var. *pumilio* | Pakistan | 1.32 | 1.84 | 1.55 | 0.99 | 1.42 |
| 158 | CGN13253 | *P. sativum* subsp. *sativum* var. *arvense* | Ethiopia | 1.64 | 3.49 | 1.77 | 3.52 | 2.61 |
| 159 | CGN16640 | *P. sativum* subsp. *jomardii* | Sudan | 0.26 | 0.66 | 0.98 | 0.74 | 0.66 |
| 160 | CGN16562 | *P. sativum* subsp. *elatius* var. *pumilio* | Mongolia | 1.77 | 1.20 | 1.98 | 2.02 | 1.74 |
| 161 | CGN16571 | *P. sativum* subsp. *jomardii* | Egypt | 0.44 | 0.60 | 0.33 | 0.66 | 0.50 |
| 162 | CGN16581 | *P. sativum* subsp. *jomardii* | Afganistan | 0.88 | 1.48 | 1.47 | 1.10 | 1.23 |
| 163 | CGN16639 | *P. sativum* subsp. *sativum* var. *arvense* | Ethiopia | 0.68 | 1.83 | 1.23 | 1.22 | 1.24 |
| 164 | CGN16679 | *P. sativum* subsp. *jomardii* | Russia | 1.80 | 1.31 | 2.03 | 0.88 | 1.50 |
| 165 | CGN16582 | *P. sativum* subsp. *elatius* var. *pumilio* | Nepal | 0.80 | 0.34 | 0.69 | 0.64 | 0.62 |
| 166 | CGN16684 | *P. sativum* subsp. *jomardii* | Greece | 0.52 | 0.12 | 0.59 | 0.90 | 0.53 |
| 167 | CGN16646 | *P. sativum* subsp. *elatius* var. *pumilio* | Mongolia | 0.70 | 1.53 | 1.46 | 1.24 | 1.23 |
| 168 | CGN16636 | *P. abyssinicum* | Ethiopia | 0.91 | 0.77 | 0.34 | 1.03 | 0.76 |
| 169 | CGN03328 | *P. sativum* subsp. *elatius* var. *pumilio* | Pakistan | 0.74 | 0.64 | 1.35 | 0.68 | 0.85 |
| 170 | CGN03170 | *P. sativum* subsp. *sativum* var. *arvense* | Irak | 2.04 | 2.26 | 3.08 | 2.66 | 2.51 |
| 171 | CGN03190 | *P. sativum* subsp. *jomardii* | Turkey | 1.43 | 3.46 | 2.10 | 2.64 | 2.41 |
| 172 | CGN03245 | *P. sativum* subsp. *sativum* var. *arvense* | Ethiopia | 3.78 | 1.73 | 1.50 | 3.48 | 2.62 |
| 173 | CGN03165 | *P. sativum* subsp. *jomardii* | Turkey | 1.09 | 3.40 | 2.18 | 3.04 | 2.43 |
| 174 | CGN03289 | *P. sativum* subsp. *elatius* var. *pumilio* | Pakistan | 2.86 | 1.97 | 1.86 | 1.89 | 2.14 |
| 175 | CGN03171 | *P. sativum* subsp. *jomardii* | Turkey | 1.42 | 2.43 | 6.52 | 2.14 | 3.13 |
| 176 | CGN03290 | *P. sativum* subsp. *elatius* var. *pumilio* | Pakistan | 1.08 | 0.49 | 0.83 | 1.01 | 0.85 |
| 177 | CGN03305 | *P. sativum* subsp. *elatius* var. *pumilio* | Pakistan | 1.32 | 2.20 | 2.54 | 2.27 | 2.09 |
| 178 | CGN02921 | *P. sativum* subsp. *sativum* var. *sativum* | Italy | 0.67 | 2.49 | 1.55 | 2.19 | 1.73 |
| 179 | CGN03003 | *P. sativum* subsp. *sativum* var. *sativum* | France | 0.04 | 0.15 | 0.23 | 0.56 | 0.24 |
| 180 | CGN03273 | *P. sativum* subsp. *sativum* var. *arvense* | Peru | 2.33 | 2.54 | 1.25 | 1.93 | 2.01 |
| 181 | CGN03229 | *P. sativum* subsp. *sativum* var. *arvense* | Ethiopia | 2.50 | 1.60 | 3.05 | 3.12 | 2.56 |
| 182 | PI 413686 | *P. sativum* subsp. *sativum* var. *sativum* | Hungary | 3.82 | 3.32 | 2.82 | 3.11 | 3.27 |
| 183 | PI 477371 | *P. sativum* subsp. *jomardii* | Denmark | 1.30 | 1.38 | 1.01 | 1.03 | 1.18 |
| 184 | PI 307666 | *P. sativum* subsp. *sativum* var. *arvense* | Costa Rica | 1.29 | 1.60 | 1.02 | 3.39 | 1.82 |
| 185 | PI 307666 | *P. sativum* subsp. *jomardii* | Costa Rica | 1.77 | 1.87 | 2.60 | 2.80 | 2.26 |
| 186 | PI 324693 | *P. sativum* subsp. *sativum* var. *sativum* | Hungary | 2.26 | 5.21 | 1.32 | 2.26 | 2.76 |
| 187 | PI 324705 | *P. sativum* subsp. *jomardii* | France | 2.33 | 1.98 | 3.18 | 2.44 | 2.48 |
| 188 | PI 355905 | *P. sativum* subsp. *sativum* var. *sativum* | Japan | 2.69 | 2.13 | 2.70 | 2.89 | 2.60 |
| 189 | PI 241593 | *P. sativum* subsp. *sativum* var. *arvense* | Taiwan | 0.57 | 1.59 | 1.42 | 3.28 | 1.71 |
| 190 | PI 273207 | *P. sativum* subsp. *elatius* var. *elatius* | Bulgaria | 0.14 | 0.10 | 0.36 | 0.33 | 0.23 |
| 191 | PI 266070 | *P. sativum* subsp. *jomardii* | Sweden | 2.68 | 2.25 | 3.31 | 4.51 | 3.19 |
| 192 | PI 198074 | *P. sativum* subsp. *jomardii* | Sweden | 0.80 | 1.31 | 0.85 | 2.67 | 1.41 |
| 193 | PI 357292 | *P. sativum* subsp. *sativum* var. *sativum* | North Macedonia | 0.46 | 0.46 | 0.77 | 0.78 | 0.61 |
| 194 | PI 357293 | *P. sativum* subsp. *sativum* var. *sativum* | North Macedonia | 0.99 | 0.73 | 0.58 | 1.07 | 0.84 |
| 195 | PI 249645 | *P. sativum* subsp. *sativum* var. *arvense* | India | 1.17 | 1.27 | 1.77 | 1.60 | 1.45 |
| 196 | PI 357048 | *P. sativum* subsp. *elatius* var. *pumilio* | India | 0.81 | 0.95 | 0.74 | 1.20 | 0.93 |
| 197 | PI 357289 | *P. sativum* subsp. *sativum* var. *sativum* | North Macedonia | 0.27 | 1.56 | 0.26 | 1.76 | 0.97 |
| 198 | PI 253968 | *P. sativum* subsp. *elatius* var. *pumilio* | Afganistan | 0.37 | 1.01 | 1.24 | 0.71 | 0.83 |
| 199 | PI 103058 | *P. sativum* subsp. *sativum* var. *sativum* | China | 1.20 | 1.26 | 0.74 | 1.15 | 1.09 |
| 200 | PI 180329 | *P. sativum* subsp. *jomardii* | India | 0.03 | 0.31 | 0.24 | 1.22 | 0.45 |
| 201 | PI 184131 | *P. sativum* subsp. *sativum* var. *sativum* | Serbia | 1.49 | 1.97 | 2.10 | 2.44 | 2.00 |
| 202 | PI 124478 | *P. sativum* subsp. *sativum* var. *arvense* | Pakistan | 1.32 | 2.49 | 2.35 | 2.04 | 2.05 |
| 203 | PI 124479 | *P. sativum* subsp. *sativum* var. *sativum* | Pakistan | 0.88 | 2.41 | 1.66 | 2.07 | 1.75 |
| 204 | PI 124479 | *P. sativum* subsp. *sativum* var. *arvense* | Pakistan | 0.67 | 2.85 | 0.71 | 3.01 | 1.81 |
| 205 | JI 2480 | *P. sativum* subsp. *sativum* var. *arvense* | Peru | 2.46 | 5.04 | 4.85 | 2.69 | 3.76 |
| 206 | JI 1951 | *P. sativum* subsp. *sativum* var. *arvense* | China | 2.27 | 1.79 | 0.80 | 1.76 | 1.66 |
| 207 | JI 2302 | *P. sativum* subsp. *sativum* var. *sativum* | Sweden | 2.12 | 1.84 | 1.21 | 2.63 | 1.95 |
| 208 | JI 1566 | *P. sativum* subsp. *sativum* var. *sativum* | USA | 1.57 | 0.64 | 1.18 | 1.46 | 1.21 |
| 209 | PI 608038 | *P. sativum* subsp. *sativum* var. *sativum* | USA | 3.19 | 3.12 | 2.87 | 2.80 | 3.00 |
| 210 | PI 613100 | *P. sativum* subsp. *sativum* var. *sativum* | USA | 1.41 | 0.96 | 1.03 | 1.61 | 1.25 |
| 211 | Atc-4235-53 | *P. sativum* subsp. *elatius* var. *pumilio* | Australia | 0.19 | 5.17 | 1.31 | 3.34 | 2.51 |
| 212 | Boreen | *P. sativum* subsp. *sativum* var. *sativum* | Australia | 0.98 | 2.93 | 1.83 | 1.52 | 1.82 |
| 213 | Danclale | *P. sativum* subsp. *jomardii* | Australia | 5.30 | 2.07 | 2.85 | 6.42 | 4.16 |
| 214 | Kagpa | *P. sativum* subsp. *sativum* var. *sativum* | Australia | 4.19 | 2.19 | 2.68 | 4.69 | 3.44 |
| 215 | M5 | *P. sativum* subsp. *sativum* var. *sativum* | Australia | 0.63 | 1.24 | 1.97 | 1.81 | 1.41 |
| 216 | Pinochio | *P. sativum* subsp. *sativum* var. *sativum* | Denmark | 0.67 | 0.81 | 1.23 | 1.17 | 0.97 |
| 217 | B 99-114 | *P. sativum* subsp. *sativum* var. *sativum* | Czech Republic | 1.22 | 1.60 | 1.00 | 2.09 | 1.48 |
| 218 | AGT 205,21 | *P. sativum* subsp. *sativum* var. *sativum* | Czech Republic | 1.42 | 1.79 | 0.78 | 1.89 | 1.47 |
| 219 | Morris | *P. sativum* subsp. *sativum* var. *sativum* | Czech Republic | 0.59 | 1.36 | 1.35 | 2.05 | 1.34 |
| 220 | JI 1210 | *P. sativum* subsp. *sativum* var. *sativum* | France | 1.02 | 1.83 | 1.44 | 1.86 | 1.54 |
| 221 | JI 1412 | *P. sativum* subsp. *sativum* var. *sativum* | USA | 0.42 | 2.16 | 1.90 | 2.31 | 1.70 |
| 222 | JI 1559 | *P. sativum* subsp. *sativum* var. *arvense* | Mexico | 1.14 | 2.31 | 2.37 | 4.62 | 2.61 |
| 223 | JI 1747 | *P. sativum* subsp. *sativum* var. *sativum* | Germany | 1.97 | 1.20 | 1.54 | 1.01 | 1.43 |
| 224 | JI 1760 | *P. sativum* subsp. *sativum* var. *sativum* | UK | 0.56 | 1.28 | 0.18 | 1.65 | 0.92 |
| 225 | JI 210 | *P. sativum* subsp. *sativum* var. *sativum* | India | 0.58 | 0.12 | 0.54 | 1.26 | 0.63 |
| 226 | JI 252 | *P. sativum* subsp. *elatius* var. *pumilio* | Ethiopia | 0.60 | 0.40 | 0.54 | 1.32 | 0.72 |
| 227 | JI 82 | *P. sativum* subsp. *elatius* var. *pumilio* | Afganistan | 0.57 | 0.59 | 0.33 | 1.30 | 0.70 |
| 228 | Messire | *P. sativum* subsp. *sativum* var. *sativum* | France | 1.03 | 1.76 | 0.98 | 2.48 | 1.56 |
| 229 | Radley | *P. sativum* subsp. *sativum* var. *sativum* | UK | 1.24 | 2.56 | 1.63 | 1.84 | 1.82 |
| 230 | Ballet | *P. sativum* subsp. *sativum* var. *sativum* | UK | 0.50 | 1.66 | 2.61 | 2.22 | 1.75 |
| 231 | W6 17515 | *P. sativum* subsp. *sativum* var. *sativum* | USA | 0.53 | 0.57 | 0.14 | 0.83 | 0.52 |
| 232 | W6 17516 | *P. sativum* subsp. *sativum* var. *sativum* | USA | 1.85 | 2.00 | 1.09 | 2.49 | 1.86 |
| 233 | W6 17517 | *P. sativum* subsp. *sativum* var. *sativum* | USA | 2.63 | 2.10 | 1.23 | 5.47 | 2.86 |
| 234 | W6 17518 | *P. sativum* subsp. *sativum* var. *sativum* | USA | 0.85 | 1.86 | 1.35 | 2.11 | 1.54 |
| 235 | W6 17520 | *P. sativum* subsp. *sativum* var. *sativum* | USA | 1.51 | 4.31 | 3.14 | 3.48 | 3.11 |
| 236 | KEBBY | *P. sativum* subsp. *sativum* var. *sativum* | UK | 0.43 | 0.75 | 0.41 | 1.02 | 0.65 |
| 237 | POLAR | *P. sativum* subsp. *sativum* var. *sativum* | Spain | 0.83 | 1.32 | 1.09 | 1.97 | 1.30 |
| 238 | W6 17519 | *P. sativum* subsp. *sativum* var. *sativum* | USA | 0.97 | 1.36 | 2.15 | 2.48 | 1.74 |
| 239 | W6 17521 | *P. sativum* subsp. *sativum* var. *sativum* | USA | 1.30 | 1.14 | 1.14 | 1.35 | 1.23 |
| 240 | BGE023667 | *P. sativum* subsp. *jomardii* | Spain | 1.93 | 3.66 | 7.84 | 3.03 | 4.11 |
| 241 | BGE025727 | *P. sativum* subsp. *jomardii* | Spain | 4.00 | 5.66 | 5.48 | 5.69 | 5.21 |
| 242 | PI 358608 | *P. sativum* subsp. *sativum* var. *arvense* | Ethiopia | 0.62 | 1.51 | 1.48 | 0.99 | 1.15 |
| 243 | PI 358609 | *P. abyssinicum* | Ethiopia | 0.11 | 0.00 | 0.12 | 0.69 | 0.23 |
| 244 | PI 173055 | *P. sativum* subsp. *elatius* var. *elatius* | Turkey | 0.29 | 0.24 | 0.12 | 0.15 | 0.20 |
| 245 | PI 120617 | *P. sativum* subsp. *elatius* var. *elatius* | Turkey | 0.19 | 0.00 | 0.27 | 0.05 | 0.13 |
| 246 | PI 273209 | *P. sativum* subsp. *elatius* var. *elatius* | Russia | 0.48 | 0.87 | 0.46 | 1.02 | 0.71 |
| 247 | PI 344003 | *P. sativum* subsp. *sativum* var. *arvense* | Turkey | 3.18 | 3.50 | 3.09 | 4.21 | 3.49 |
| 248 | PI 344005 | *P. sativum* subsp. *elatius* var. *elatius* | Greece | 0.52 | 0.20 | 0.13 | 1.15 | 0.50 |
| 249 | PI 344006 | *P. sativum* subsp. *elatius* var. *elatius* | Greece | 0.26 | 0.27 | 0.14 | 3.44 | 1.03 |
| 250 | PI 343976 | *P. sativum* subsp. *elatius* var. *elatius* | Turkey | 0.19 | 0.44 | 0.03 | 1.21 | 0.47 |
| 251 | PI 505059 | *P. sativum* subsp. *jomardii* | Sudan | 0.19 | 0.14 | 0.18 | 0.30 | 0.20 |
| 252 | PI 344010 | *P. sativum* subsp. *elatius* var. *elatius* | Greece | 0.00 | 0.36 | 0.15 | 0.54 | 0.26 |
| 253 | PI 344011 | *P. sativum* subsp. *elatius* var. *elatius* | Greece | 0.16 | 0.09 | 0.84 | 0.32 | 0.36 |
| 254 | PI 344013 | *P. sativum* subsp. *elatius* var. *elatius* | Greece | 0.21 | 0.12 | 0.46 | 0.37 | 0.29 |
| 255 | PI 116056 | *P. sativum* subsp. *sativum* var. *arvense* | India | 0.96 | 2.00 | 1.19 | 2.67 | 1.71 |
| 256 | PI 505127 | *P. sativum* subsp. *jomardii* | Albania | 1.39 | 4.91 | 5.06 | 2.61 | 3.49 |
| 257 | PI 242027 | *P. sativum* subsp. *jomardii* | Denmark | 0.11 | 0.08 | 0.59 | 0.43 | 0.30 |
| 258 | PI 269762 | *P. sativum* subsp. *jomardii* | UK | 0.06 | 0.05 | 0.90 | 0.56 | 0.39 |
| 259 | PI 343987 | *P. sativum* subsp. *sativum* var. *arvense* | Turkey | 1.88 | 2.50 | 1.31 | 1.56 | 1.81 |
| 260 | PI 505080 | *P. sativum* subsp. *jomardii* | Cyprus | 3.76 | 5.82 | 2.81 | 3.06 | 3.86 |
| 261 | PI 505111 | *P. sativum* subsp. *jomardii* | Syria | 1.23 | 3.58 | 4.32 | 3.11 | 3.06 |
| 262 | PI 268480 | *P. sativum* subsp. *elatius* var. *pumilio* | Afganistan | 0.53 | 1.29 | 1.73 | 1.81 | 1.34 |
| 263 | JI 45 | *P. sativum* subsp. *jomardii* | Georgia | 1.18 | 1.18 | 1.06 | 1.15 | 1.14 |
| 264 | JI 198 | *P. sativum* subsp. *sativum* var. *arvense* | Israel | 1.77 | 1.53 | 2.37 | 1.74 | 1.85 |
| 265 | JI 199 | *P. sativum* subsp. *sativum* var. *arvense* | Israel | 1.74 | 0.68 | 1.13 | 1.46 | 1.25 |
| 266 | JI 225 | *P. abyssinicum* | Ethiopia | 0.68 | 0.00 | 0.94 | 0.97 | 0.65 |
| 267 | JI 227 | *P. abyssinicum* | Ethiopia | 0.16 | 0.19 | 0.17 | 0.51 | 0.26 |
| 268 | JI 241 | *P. sativum* subsp. *elatius* var. *pumilio* | Israel | 1.12 | 1.04 | 0.16 | 2.05 | 1.09 |
| 269 | JI 254 | *P. sativum* subsp. *elatius* var. *elatius* | Ethiopia | 1.11 | 0.20 | 0.65 | 0.38 | 0.59 |
| 270 | JI 804 | *P. sativum* subsp. *elatius* var. *pumilio* | unknown | 0.56 | 1.76 | 0.91 | 1.04 | 1.07 |
| 271 | JI 1398 | *P. sativum* subsp. *elatius* var. *pumilio* | China | 0.97 | 2.70 | 1.15 | 1.28 | 1.53 |
| 272 | JI 1428 | *P. sativum* subsp. *elatius* var. *pumilio* | China | 1.44 | 0.00 | 1.17 | 1.48 | 1.02 |
| 273 | JI 1854 | *P. sativum* subsp. *elatius* var. *pumilio* | Israel | 1.93 | 1.98 | 2.25 | 2.57 | 2.18 |
| 274 | JI 2116 | *P. sativum* subsp. *sativum* var. *arvense* | Spain | 1.50 | 6.18 | 2.55 | 4.39 | 3.66 |
| 275 | JI 2202 | *P. abyssinicum* | Yemen | 0.72 | 0.02 | 0.95 | 0.29 | 0.49 |
| 276 | PIS 1318/91 | *P. sativum* subsp. *elatius* var. *elatius* | Israel | 0.00 | 0.00 | 0.00 | 0.04 | 0.01 |
| 277 | CGN10205 | *P. sativum* subsp. *elatius* var. *elatius* | Turkey | 0.33 | 1.02 | 1.05 | 1.01 | 0.85 |
| 278 | CGN10206 | *P. sativum* subsp. *elatius* var. *elatius* | Lebanon | 0.39 | 0.43 | 0.72 | 0.73 | 0.57 |
| 279 | CGN10193 | *P. sativum* subsp. *jomardii* | unknown | 0.48 | 0.67 | 0.97 | 1.99 | 1.03 |
| 280 | IFPI 3365 | *P. sativum* subsp. *sativum* var. *arvense* | Turkey | 0.92 | 1.79 | 2.49 | 3.01 | 2.05 |
| 281 | IFPI 3370 | *P. sativum* subsp. *jomardii* | Turkey | 1.34 | 2.61 | 3.12 | 3.54 | 2.65 |
| 282 | IFPI 387 | *P. sativum* subsp. *jomardii* | USSR | 2.91 | 2.19 | 3.80 | 3.78 | 3.17 |
| 283 | IFPI 436 | *P. sativum* subsp. *jomardii* | Egypt | 0.25 | 0.48 | 0.96 | 1.80 | 0.87 |
| 284 | IFPI 2348 | *P. sativum* subsp. *sativum* var. *arvense* | Ethiopia | 1.23 | 1.67 | 2.90 | 1.98 | 1.95 |
| 285 | IFPI 2350 | *P. sativum* subsp. *sativum* var. *arvense* | Ethiopia | 1.58 | 2.27 | 1.72 | 2.35 | 1.98 |
| 286 | IFPI 2351 | *P. sativum* subsp. *sativum* var. *arvense* | Ethiopia | 2.15 | 3.63 | 2.79 | 2.80 | 2.84 |
| 287 | IFPI 2352 | *P. sativum* subsp. *sativum* var. *arvense* | Ethiopia | 1.60 | 3.40 | 2.21 | 2.03 | 2.31 |
| 288 | IFPI 2353 | *P. sativum* subsp. *sativum* var. *arvense* | Ethiopia | 0.91 | 1.35 | 1.57 | 2.32 | 1.54 |
| 289 | IFPI 2354 | *P. sativum* subsp. *sativum* var. *arvense* | Ethiopia | 2.44 | 1.43 | 2.63 | 2.39 | 2.22 |
| 290 | IFPI 2356 | *P. sativum* subsp. *sativum* var. *arvense* | Ethiopia | 1.26 | 1.66 | 1.28 | 1.54 | 1.44 |
| 291 | IFPI 2357 | *P. sativum* subsp. *sativum* var. *arvense* | Ethiopia | 1.69 | 2.19 | 1.23 | 1.78 | 1.72 |
| 292 | IFPI 2358 | *P. sativum* subsp. *sativum* var. *arvense* | Ethiopia | 2.17 | 1.40 | 0.96 | 2.07 | 1.65 |
| 293 | IFPI 2360 | *P. sativum* subsp. *sativum* var. *arvense* | Ethiopia | 1.11 | 0.92 | 1.09 | 1.35 | 1.12 |
| 294 | IFPI 2362 | *P. sativum* subsp. *sativum* var. *arvense* | Ethiopia | 0.94 | 2.96 | 0.73 | 1.93 | 1.64 |
| 295 | IFPI 2363 | *P. sativum* subsp. *sativum* var. *arvense* | Ethiopia | 0.49 | 0.77 | 1.72 | 2.89 | 1.47 |
| 296 | IFPI 2364 | *P. sativum* subsp. *sativum* var. *arvense* | Ethiopia | 1.43 | 2.61 | 2.47 | 2.10 | 2.15 |
| 297 | IFPI 2365 | *P. sativum* subsp. *sativum* var. *arvense* | Ethiopia | 1.29 | 1.12 | 1.43 | 1.87 | 1.43 |
| 298 | IFPI 2367 | *P. sativum* subsp. *sativum* var. *arvense* | Ethiopia | 0.60 | 0.87 | 0.48 | 1.75 | 0.92 |
| 299 | IFPI 2369 | *P. sativum* subsp. *sativum* var. *arvense* | Ethiopia | 1.78 | 2.49 | 1.63 | 1.59 | 1.87 |
| 300 | IFPI 2370 | *P. sativum* subsp. *sativum* var. *arvense* | Ethiopia | 0.35 | 0.83 | 1.19 | 3.13 | 1.38 |
| 301 | IFPI 2371 | *P. sativum* subsp. *sativum* var. *arvense* | Ethiopia | 1.37 | 1.38 | 3.03 | 1.90 | 1.92 |
| 302 | IFPI 2372 | *P. sativum* subsp. *sativum* var. *arvense* | Ethiopia | 1.84 | 2.04 | 2.31 | 1.88 | 2.02 |
| 303 | IFPI 2441 | *P. sativum* subsp. *jomardii* | Denmark | 0.05 | 0.42 | 0.92 | 0.98 | 0.59 |
| 304 | IFPI 2495 | *P. sativum* subsp. *jomardii* | UK | 0.01 | 0.69 | 0.23 | 0.80 | 0.43 |
| 305 | IFPI 3232 | *P. fulvum* | Syria | 0.10 | 0.00 | 0.06 | 0.00 | 0.04 |
| 306 | IFPI 3250 | *P. sativum* subsp. *jomardii* | Syria | 0.35 | 0.60 | 1.08 | 1.03 | 0.76 |
| 307 | IFPI 3252 | *P. sativum* subsp. *elatius* var. *elatius* | Syria | 0.77 | 0.23 | 0.22 | 0.10 | 0.33 |
| 308 | IFPI 3253 | *P. fulvum* | Syria | 0.00 | 0.00 | 0.03 | 0.00 | 0.01 |
| 309 | IFPI 3257 | *P. fulvum* | Syria | 0.00 | 0.07 | 0.30 | 0.23 | 0.15 |
| 310 | IFPI 3260 | *P. fulvum* | Syria | 0.03 | 0.00 | 0.03 | 2.29 | 0.58 |
| 311 | IFPI 3261 | *P. fulvum* | Syria | 0.00 | 0.00 | 0.05 | 0.00 | 0.01 |
| 312 | IFPI 3262 | *P. fulvum* | Syria | 0.00 | 0.00 | 0.07 | 0.19 | 0.06 |
| 313 | IFPI 3280 | *P. sativum* subsp. *elatius* var. *elatius* | Syria | 0.55 | 0.04 | 0.33 | 3.08 | 1.00 |
| 314 | IFPI 3282 | *P. sativum* subsp. *elatius* var. *elatius* | Syria | 0.00 | 0.11 | 0.22 | 0.00 | 0.08 |
| 315 | IFPI 3330 | *P. sativum* subsp. *jomardii* | Turkey | 0.18 | 0.29 | 1.02 | 1.40 | 0.72 |
| 316 | IFPI 3334 | *P. sativum* subsp. *elatius* var. *elatius* | Turkey | 0.08 | 0.03 | 0.00 | 0.08 | 0.05 |
| 317 | IFPI 3338 | *P. sativum* subsp. *elatius* var. *elatius* | Turkey | 0.09 | 0.16 | 0.00 | 0.03 | 0.07 |
| 318 | IFPI 3358 | *P. sativum* subsp. *jomardii* | Turkey | 0.03 | 0.03 | 0.03 | 0.05 | 0.03 |
| 319 | P691 | *P. sativum* subsp. *sativum* var. *arvense* | unknown | 0.83 | 0.67 | 0.90 | 1.48 | 0.97 |
| 320 | JI 1006 | *P. fulvum* | Israel | 0.00 | 0.17 | 0.59 | 0.20 | 0.24 |
| 321 | Cartouche | *P. sativum* subsp. *sativum* var. *sativum* | France | 0.11 | 2.29 | 2.14 | 2.05 | 1.65 |
| 322 | JI 45 | *P. sativum* subsp. *jomardii* | Georgia | 1.40 | 0.98 | 1.10 | 1.40 | 1.22 |
| 323 | Livia | *P. sativum* subsp. *sativum* var. *sativum* | Germany | 1.33 | 1.49 | 1.54 | 1.74 | 1.52 |
| 324 | Audit | *P. sativum* subsp. *sativum* var. *sativum* | France | 0.46 | 3.04 | 1.05 | 4.01 | 2.14 |
